# Fatty acid amide hydrolase inhibition for treatment of amyotrophic lateral sclerosis

**DOI:** 10.1172/jci.insight.198842

**Published:** 2026-06-11

**Authors:** Daisuke Ito, Madoka Iida, Yohei Iguchi, Atsushi Hashizume, Shinichiro Yamada, Yoshiyuki Kishimoto, Shota Komori, Kazuki Obara, Shuto Nishisaki, Satoshi Yokoi, Teppei Shimamura, Yuto Takemoto, Masahiro Nakatochi, Tomohiro Akashi, Kunihiko Hinohara, Hyeon-Cheol Lee-Okada, Yohei Okada, Junichi Niwa, Gen Sobue, Shinji Tanaka, Ken Takashina, Takehiko Yokomizo, Masahisa Katsuno

**Affiliations:** 1 Department of Neurology,; 2 Department of Clinical Research Education,; 3 Department of Pathophysiological Laboratory Sciences,; 4 Division of Systems Biology,; 5 Public Health Informatics Unit, Department of Integrated Health Sciences, and; 6 Department of Immunology, Nagoya University Graduate School of Medicine, Nagoya, Aichi, Japan.; 7 Institute for Advanced Research, Nagoya University, Nagoya, Aichi, Japan.; 8 Department of Biochemistry, Juntendo University Graduate School of Medicine, Bunkyo-ku, Tokyo, Japan.; 9 Department of Neural iPSC Research Institute for Medical Science of Aging, Aichi Medical University, Nagakute, Aichi, Japan.; 10 Department of Neurology and; 11 Aichi Medical University School of Medicine, Nagakute, Aichi, Japan.; 12 Sohyaku, Innovative Research Division, Mitsubishi Tanabe Pharma Corporation, Yokohama, Japan.

**Keywords:** Metabolism, Neuroscience, ALS, Biomarkers, Drug therapy

## Abstract

Amyotrophic lateral sclerosis (ALS) is a devastating neurodegenerative disease caused by the selective loss of upper and lower motor neurons. There is a considerable variability in the disease progression of sporadic ALS, but the molecular basis for phenotypic heterogeneity remains largely unknown. Patients with ALS often manifest systemic metabolic abnormalities such as glucose intolerance and hypermetabolic state. We conducted reverse translational research to explore therapeutic targets in ALS based on the systemic metabolic alterations in patients and identified several metabolites associated with the disease progression, including metabolites involved in the expanded endocannabinoid system (ECS). In particular, the levels of *N*-acyl taurines (NATs) were correlated with the longitudinal change in the revised ALS functional rating scale and survival. Experiments with ALS cellular models and induced pluripotent stem (iPS) cells derived from patients with ALS and SOD1^G93A^ transgenic mice revealed that PF-04457845, a fatty acid amide hydrolase inhibitor, upregulated the expanded ECS, particularly the levels of NATs and ameliorated motor neuron degeneration through the regulation of microglial environment, synapse plasticity, and neuronal development. These results collectively indicate that dysregulation of NATs is associated with ALS progression and PF-04457845 may represent a potential disease-modifying therapy for ALS.

## Abstract 2

Serum N-acyl taurines are potential prognostic biomarkers in ALS, and inhibition of fatty acid amide hydrolase (FAAH) represents a promising therapeutic strategy for the disease.

## Graphical abstract

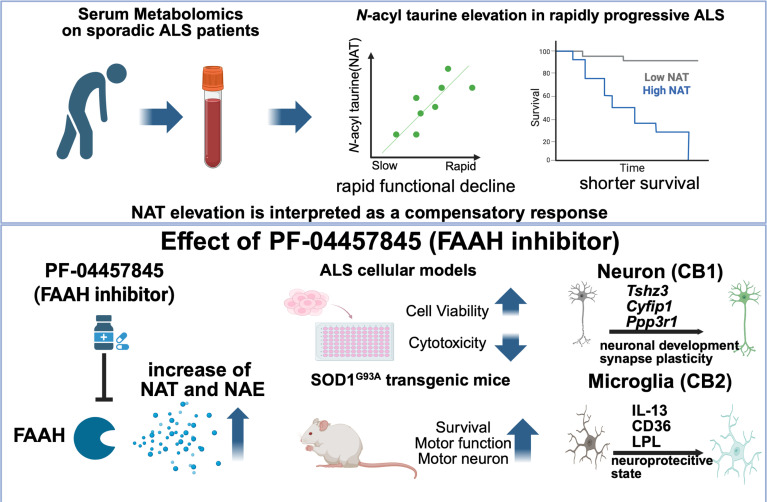

## Introduction

Amyotrophic lateral sclerosis (ALS) is a devastating neurodegenerative disease caused by the selective loss of upper and lower motor neurons. Approximately 95% of instances of ALS are sporadic, and 5% are familial. Causative genes for familial ALS are diverse, including *superoxide dismutase 1* (*SOD1*), *TARDBP*, and *C9ORF72* (1–7). A *SOD1*-based murine model has been widely used for the preclinical study of ALS therapeutic strategies because it simulates the rapid progression of motor neuron degeneration and glial cell changes characteristic of human ALS pathology. In sporadic ALS, muscle weakness and atrophy rapidly progress, and the time from onset to death is around 3–5 years, mainly as a result of respiratory failure (8, 9). However, it is widely known that there is a considerable variability in the disease progression of sporadic ALS. This variability suggests that biological factors governing disease progression may be a therapeutic target of sporadic ALS, but the molecular basis for ALS phenotypic heterogeneity remains elusive.

A great number of therapeutics for ALS that showed efficacy in preclinical studies failed in clinical trials (10, 11). Although riluzole and edaravone have been approved for ALS treatment, their efficacies are limited (12, 13). These difficulties in therapy development are, at least partially, due to the discrepancy between the molecular changes in human sporadic ALS and those in cell or animal models established by implementing familial ALS gene mutations; the cell and animal models expressing the causative gene mutations of familial ALS do not necessarily reflect the pathological mechanism of human sporadic ALS. Therefore, the development of therapeutics should be based on human ALS pathophysiology. An approach to overcome these struggles is the utilization of induced pluripotent stem (iPS) cells derived from human patients with ALS (14). Indeed, drug screening using motor neurons differentiated from iPS cells identified several candidates for ALS therapeutics (15, 16). However, this approach alone is insufficient to target glial pathology, which plays fundamental roles in ALS progression (17–20). Thus, more comprehensive approaches are needed to achieve reverse translational therapy development based on human ALS pathophysiology.

Patients with sporadic ALS often develop a pathological condition called hypermetabolism, a severe weight loss due to excess resting energy expenditure, which is related to rapid progression and poor prognosis of ALS (21–23). In addition, glucose intolerance and dyslipidemia have also been reported in patients with ALS, even at a prodromal stage of disease (24, 25). Nutritional intervention with a high-calorie and/or high-fat diet suppresses disease progression in certain populations of sporadic patients with ALS (26, 27). These observations suggest a causative role of systemic metabolic changes in ALS.

Herein, we aimed to develop a therapeutic strategy for ALS with a special focus on metabolic alterations in patients by reverse translating clinical observations to basic research. We first conducted a clinical study to elucidate metabolic changes related to disease progression, screened compounds targeting the identified metabolic profiles in cellular models, and then tested a hit compound — a fatty acid amide hydrolase (FAAH) inhibitor, PF-04457845 — in motor neurons differentiated from iPS cells (iPS-MN) derived from patients with ALS and a mouse model of ALS. Our study identified that the dysregulation of *N*-acyl taurines (NATs), endogenous lipid mediators belonging to the expanded endocannabinoid system (ECS), is associated with disease progression in patients with sporadic ALS and that a pharmacological approach that activates NATs and the expanded ECS is a potential therapeutic strategy for ALS.

## Results

### Serum metabolome and lipid mediator analysis in sporadic patients with ALS.

**Figure 1:**
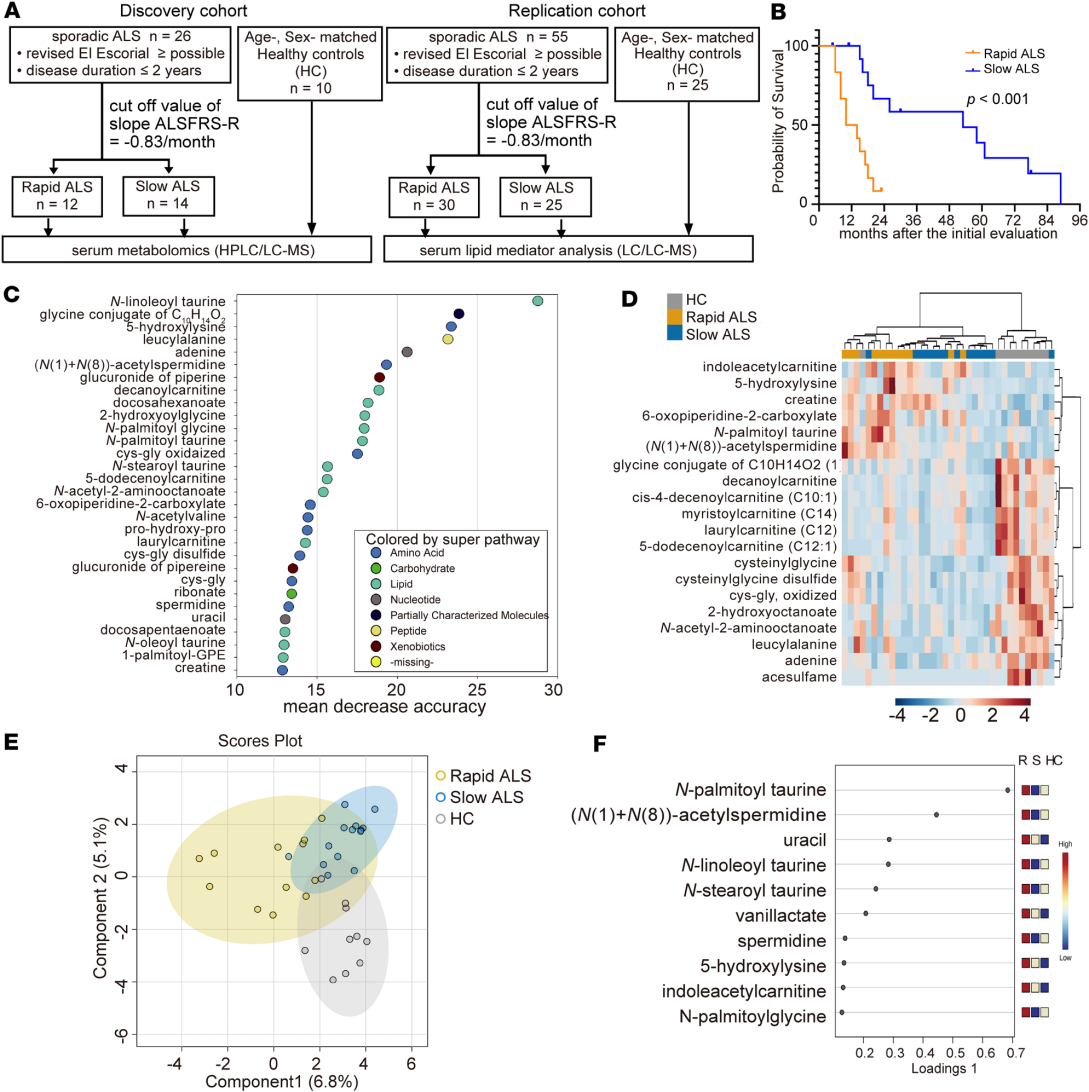
Metabolomics analysis of patients with ALS and healthy individuals. (**A**) Flow chart of metabolome analysis. For discovery cohort, we included patients with definite, probable, or possible ALS that met the revised El Escorial criteria and had disease duration less than 2 years. Patients with ALS were divided into rapidly progressive (Rapid ALS, *n* = 12) and slowly progressive (Slow ALS, *n* = 14) groups according to the median longitudinal change in ALSFRS-R (slope ALSFRS-R) from the first evaluation to 6 months. Rapid ALS was defined as a decline of ≥ 5 points in ALSFRS-R over 6 months (slope ALSFRS-R ≤ –0.83/month), and the others were classified as Slow ALS. Ten age- and sex-matched healthy controls were also included. In the replication cohort, 55 patients with ALS and 25 healthy controls were included. Patients with ALS were similarly classified using the same cut-off value (Rapid ALS, *n* = 30; Slow ALS, *n* = 25). (**B**) Kaplan-Meier curves of Rapid ALS and Slow ALS groups (median survival: 12 months vs 53 months, *P* = 0.0004, log-rank test). The primary endpoint was defined as the introduction of tracheostomy positive pressure ventilation (TPPV) or death of the patient. (**C**) Top 30 metabolites that discriminate among Rapid ALS, Slow ALS, and healthy controls by random forest classification. The metabolites were ranked according to their mean decrease accuracy. (**D**) Heatmap analysis of the top 20 metabolites identified by 1-way ANOVA. (**E**) Sparse partial least squares discriminant analysis (sPLS-DA) plots by MetaboAnalyst 6.0 to discriminate Rapid ALS, Slow ALS, and healthy controls (HC). (**F**) Loading plots of the top 10 metabolites by sPLS-DA shown in **E**.

The serum metabolome was analyzed in patients with sporadic ALS and healthy controls. A total of 26 patients with ALS and 10 healthy controls were analyzed in the discovery cohort (Figure 1A). The median longitudinal change in the revised ALS Functional Rating Scale (ALSFRS-R) at 6 months (slope ALSFRS-R) was –0.67/month (equivalent to 4-point decline over 6 months). Therefore, patients with ALS whose decline of ALSFRS-R for 6 months was more than 5 points were defined as having rapidly progressive ALS (rapid ALS, *n* = 12), and the others were defined as having slowly progressive ALS (slow ALS, *n* = 14). Baseline characteristics were not different between the patients in the Rapid ALS and Slow ALS groups (Supplemental Tables 1 and 2; supplemental material available online with this article; https://doi.org/10.1172/jci.insight.198842DS1). The Kaplan-Meier curve with the primary endpoint of the introduction of tracheostomy positive pressure ventilation (TPPV) or death clearly separated Rapid ALS and Slow ALS (Figure 1B). These findings confirmed the validity of the criteria used for categorizing patients with ALS based on the longitudinal changes in ALSFRS-R scores. Sera of patients with ALS and healthy controls were subjected to metabolome analysis using Metabolon UPLC-MS/MS.

Serum metabolomics detected 867 metabolites in total. Thirteen metabolites were significantly different among the 3 groups: Rapid ALS, Slow ALS, and healthy controls (Supplemental Figure 1 and Supplemental Table 3). Random forest analysis, whose predictive accuracy for discrimination of 3 groups was 61%, which is above random chance, showed that *N*-linoleoyl taurine, a metabolite belonging to NAT, was the most effective metabolite for distinguishing the groups (Figure 1C). NATs are taurine molecules conjugated to fatty acyl chains, and *N*-linoleoyl taurine is one representative species within this class. The top-ranked metabolites in the random forest analysis were mainly related to the expanded ECS including NATs, nucleic sugar, acylcarnitine, and polyamine metabolism. Similarly, sparse partial least squares discriminant analysis (sPLS-DA) and heatmap analysis also demonstrated that NATs, nucleic sugar, acylcarnitine, and polyamine metabolites were the discriminant metabolic factors for the 3 groups (Figure 1, D–F).

**Figure 2:**
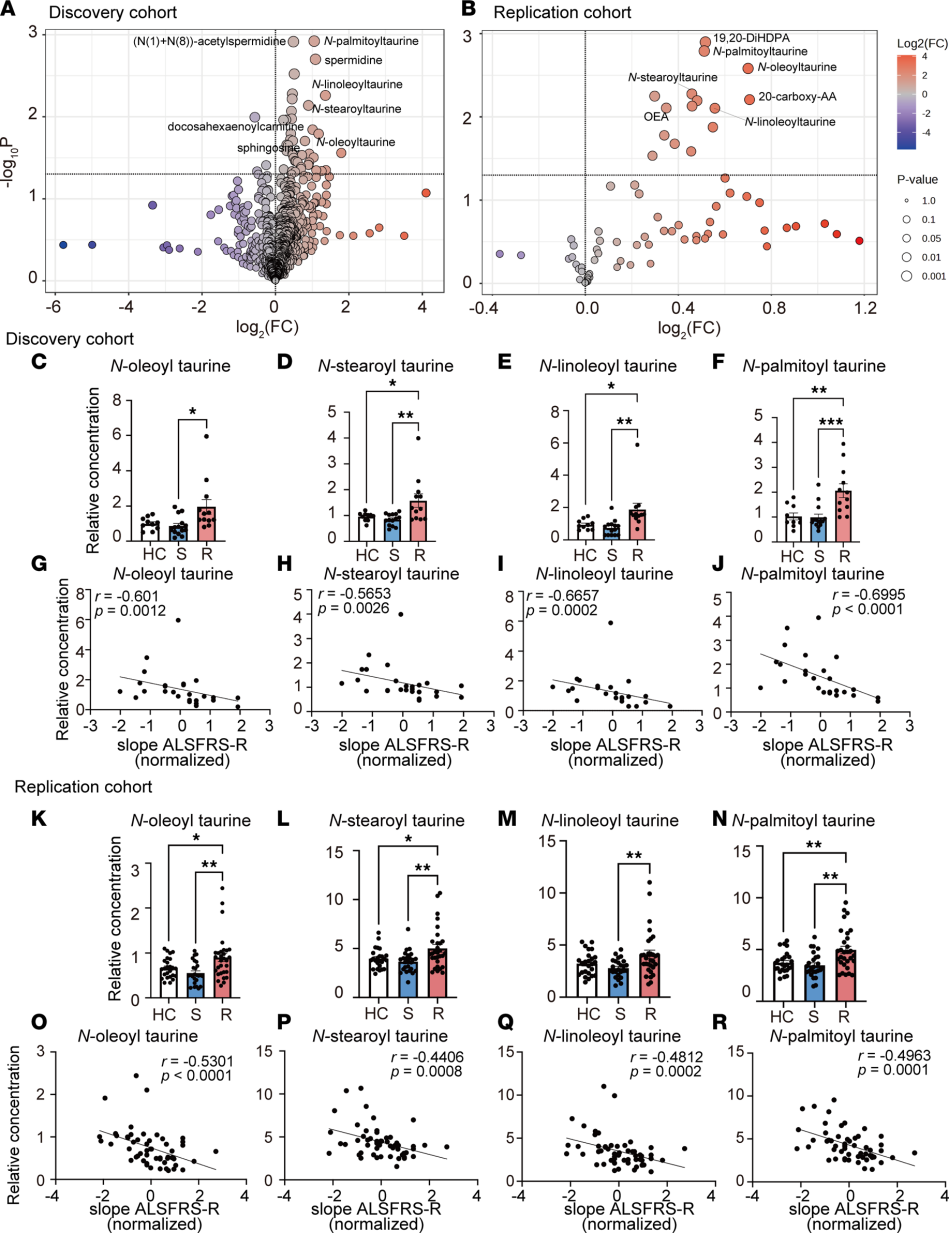
Alteration in *N*-acyl taurine metabolism among patients with rapidly progressive ALS, patients with slowly progressive ALS, and healthy individuals and their correlation with disease progression in the discovery cohort and the replication cohort. (**A**) Volcano plot of serum metabolomics analysis comparing between patients with rapid ALS and patients with slow ALS in the discovery cohort. (**B**) Volcano plot of serum lipid mediator analysis comparing between patients with rapid ALS and patients with slow ALS in the replication cohort. (**C**–**F**) Serum levels of *N*-acyl taurine metabolism in the discovery cohort (**C**, *N*-oleoyl taurine; **D**, *N*-stearoyl taurine; **E**, *N*-linoleoyl taurine; **F**, *N*-palmitoyl taurine). (**G**–**J**) Correlation between the serum levels of N-acyl taurines and the prospective, longitudinal change in ALSFRS-R between the first and second evaluations (slope ALSFRS-R) in the discovery cohort (**G**, *N*-oleoyl taurine; **H**, *N*-stearoyl taurine; **I**, *N*- linoleoyl taurine; **J**, *N*-palmitoyl taurine). (**K**–**N**) Serum levels of *N*-acyl taurine metabolism in the replication cohort (**K**, *N*-oleoyl taurine; **L**, *N*-stearoyl taurine; **M**, *N*- linoleoyl taurine; **N**, *N*-palmitoyl taurine). (**O**–**R**) Correlation between the serum levels of N-acyl taurines and slope ALSFRS-R in the replication cohort (**O**, *N*-oleoyl taurine; **P**, *N*-stearoyl taurine; **Q**, *N*-linoleoyl taurine; **R**, *N*-palmitoyl taurine). The discovery cohort was analyzed by broad untargeted metabolomics, whereas the replication cohort was analyzed using a targeted lipid mediator panel; therefore, the total number of detected analytes differed between the 2 cohorts. ALSFRS-R slope values were transformed using the Yeo-Johnson transformation before correlation analysis. One-way ANOVA and Tukey’s post hoc analysis were performed (*P < 0.05, **P < 0.01, and ***P < 0.001). HC, healthy controls; R, Rapid ALS; S, Slow ALS. A coefficient value (*r*) of 0.40–0.59 is considered moderate, while 0.60–0.79 is considered strong in Spearman’s rank correlation coefficient. Data are shown as mean ± SEM.

To identify metabolic factors relevant to the disease progression of ALS, we further performed a comparison between the Rapid ALS and Slow ALS groups. Volcano plot and heatmap analyses showed an increase in NATs and polyamine metabolites in the Rapid ALS group, and pathway enrichment analyses also showed alterations in those metabolic pathways (Figure 2A and Supplemental Figures 2–4). In addition, altered levels of bile acid metabolites were also found in the Rapid ALS group with pathway analyses (Supplemental Figure 3 and Supplemental Table 4). According to these clinical metabolome analyses, we selected 5 metabolic pathways, NATs, polyamine/arginine, xanthine, bile acid, and acylcarnitine metabolism, as candidate metabolic biomarkers associated with the progression of ALS. Detailed alterations in identified metabolic pathways among the 3 groups are presented in Supplemental Table 5.

**Figure 3:**
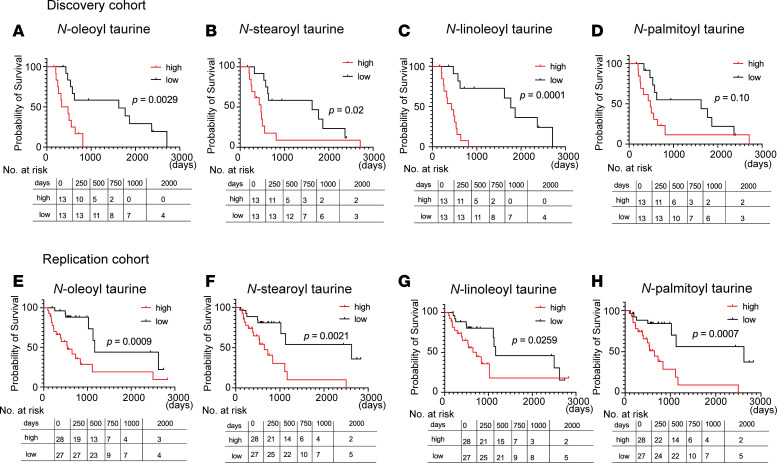
Kaplan-Meier survival curves based on serum *N*-acyl taurine levels in the discovery and replication cohorts. (**A**–**D**) Kaplan-Meier survival curves of the discovery cohort stratified by serum levels of *N*-acyl taurines: (**A**) *N*-oleoyl taurine, (**B**) *N*-stearoyl taurine, (**C**) *N*-linoleoyl taurine, and (**D**) *N*-palmitoyl taurine. (**E**–**H**) Kaplan-Meier survival curves of the replication cohort for the corresponding metabolites: (**E**) *N*-oleoyl taurine, (**F**) *N*-stearoyl taurine, (**G**) *N*-linoleoyl taurine, and (**H**) *N*-palmitoyl taurine. In both cohorts, patients were divided into high and low groups based on the median serum concentration of each *N*-acyl taurine. The primary endpoint was defined as the introduction of tracheostomy positive pressure ventilation (TPPV) or death of the patient. Log-rank test was used to compare survival between groups.

Among the identified metabolic pathways, the alterations of NATs were prominent, which belong to the expanded endocannabinoid system and act as a lipid mediators. To validate the changes in NATs, we performed a comprehensive lipid mediator analysis in the replication cohort (ALS, *n* = 55; healthy controls, *n* = 25) (Figure 1A). The smaller number of analytes detected in the replication cohort likely resulted from the use of a targeted lipid mediator validation assay, in contrast to the broad untargeted metabolomics platform used for the discovery cohort. Among the 75 lipid mediators analyzed, NAT levels were higher in the Rapid ALS group compared with the Slow ALS group, consistent with the discovery cohort, demonstrating reproducible results (Figure 2, A and B). A detailed analysis of the 4 detected NAT species revealed that, in both cohorts, their concentrations were elevated in the Rapid ALS group compared with healthy controls and the Slow ALS group. Furthermore, their levels showed a correlation with the rate of disease progression, as indicated by the slope of ALSFRS-R (Figure 2, C–R). When analyzed separately by sex, similar trends were observed in both male and female patients in both cohorts (Supplemental Figures 5 and 6). In addition, survival analyses were conducted by stratifying patients in both cohorts into 2 groups based on the median serum concentrations of each NAT. These analyses revealed that patients with higher NAT levels exhibited shorter survival durations, and this trend was consistently observed across both cohorts (Figure 3). These findings are also consistent with the observed correlations between NAT levels and disease progression rates. The other metabolites in the ECS such as *N*-acyl ethanolamines (NAE) were not altered among the 3 groups (Supplemental Figure 7).

### In vitro drug screening.

**Figure 4:**
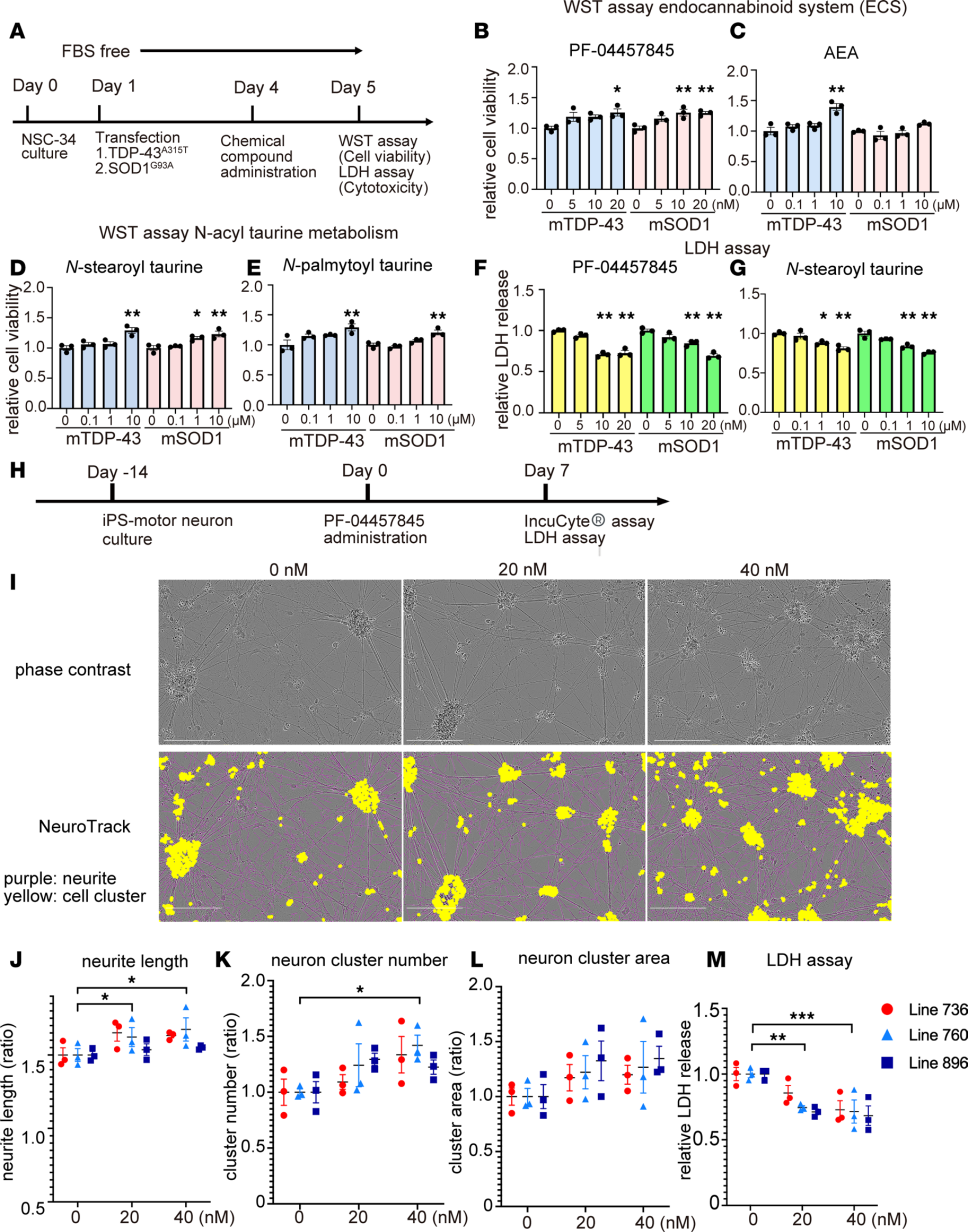
Cellular assays of compounds targeting the expanded endocannabinoid system and efficacy of PF-04457845 in NSC-34 cells and iPSC-derived motor neurons from sporadic patients with ALS. We tested a total of 29 compounds that target the identified metabolic pathways. We used NSC-34 cells transiently overexpressing TDP-43^A315T^ or SOD1^G93A^ and assessed cell viability using the WST-8 assay and cytotoxicity using the lactate dehydrogenase (LDH) assay. Results for the representative agents that modulate the expanded endocannabinoid system including *N*-acyl taurines are shown in this figure, and the other results are summarized in Supplemental Table 6. (**A**) Protocol of the cell viability assay. (**B**–**E**) Effects of compounds targeting expanded endocannabinoid system (**B**, PF-04457845; **C**, arachidonoyl ethanolamide [AEA]), and *N*-acyl taurine in expanded endocannabinoid system (**D**, *N*-stearoyl taurine; **E**, *N*-palmitoyl taurine), on cell viability. (**F** and **G**) Neuroprotective effects of PF-04457845 and *N*-stearoyl taurine were examined by LDH assays (**F**, PF-04457845; **G**, *N*-stearoyl taurine). (**H**) Protocol of administration of PF-04457845 on iPSC-derived motor neurons (iPS-MN) derived from 3 sporadic patients with ALS. (**I**) Analysis of cell morphology by IncuCyte SX5. iPS-MN were captured by phase separation and neurites (purple) and cell clusters (yellow) were automatically recognized by NeuroTrack algorithm. (**J**) The result of neurite length (*N* = 3 each from 3 iPS-MN). (**K**) The result of number of cell clusters (*N* = 3 each from 3 iPS-MN). (**L**) The result of area of cell clusters (*N* = 3 each from 3 iPS-MN). (**M**) The result of LDH assay (*N* = 3 each from 3 iPS-MN). For NSC-34 cells, 1-way ANOVA followed by Dunnett’s multiple-comparisons test was performed, with each concentration compared with the baseline condition. For iPSC-derived motor neurons, data were analyzed using a mixed-effects model, with treatment as a fixed effect and iPSC line as a random effect, followed by Dunnett’s multiple-comparisons test. (*P < 0.05, **P < 0.01, ****P < 0.0001). Scale bars: 200 μm. Data are shown as mean ± SEM.

The results of the clinical study on the relationship between the metabolome and disease progression of sporadic patients with ALS led us to drug screening using cellular models of ALS. A total of 29 chemical compounds that potentially modulate the focused metabolic pathways (the expanded ECS including NATs, polyamine/arginine, xanthine, bile acid, and acylcarnitine), together with riluzole and edaravone as controls, were administered to 2 cellular models of ALS: NSC-34 cells transiently expressing mutant TDP-43^A315T^ and those expressing mutant SOD1^G93A^ (Figure 4, A–M, and Supplemental Table 6). The 29 compounds were selected as a representative and experimentally feasible set of modulators for the progression-related metabolic pathways identified in the patient metabolomics data, with emphasis on translational relevance and suitability for human use whenever possible. Cell viability was assessed using a water-soluble tetrazolium (WST) assay. Among the tested compounds, intervention in the endocannabinoid system demonstrated the most pronounced effect. PF-04457845 and *N*-stearoyl taurine improved cell viability in both models in a concentration-dependent manner (Figure 4, B and D). Additionally, *N*-palmitoyl taurine improved cell viability in both models, although efficacy was observed at a single concentration (Figure 4E). While arachidonoyl ethanolamide (AEA, anandamide) improved the viability of NSC-34 cells expressing mutant TDP-43^A315T^, such efficacy was not verified in the cells expressing mutant SOD1^G93A^ (Figure 4C). The efficacies of PF-04457845 and *N*-stearoyl taurine were further investigated by assessing lactate dehydrogenase (LDH) release. The results demonstrated a significant reduction in LDH release (Figure 4, F and G), corroborating their potential as therapeutic agents. To evaluate its effect in a human ALS disease model, we further administered PF-04457845 to motor neurons differentiated from iPS cells (iPS-MN) derived from 3 patients with ALS (Figure 4H). Concentration-dependent neuroprotective effects were demonstrated by IncuCyte SX5 (Figure 4, I–L) and LDH assay (Figure 4M), as evidenced by preserved neurite length, number of cell clusters, and reduced LDH release. No significant line-dependent difference in treatment effect was detected among the iPSC lines. To assess disease specificity in vitro, PF-04457845 was also evaluated in mock-transfected NSC-34 cells and control iPSC-derived motor neurons; however, no significant effect of treatment was observed on WST-8 assay or LDH release in mock-transfected NSC-34 cells — nor was there a significant effect of treatment observed on neuronal morphology or LDH release in control iPSC-derived motor neurons (Supplemental Figures 8 and 9). These data suggest that PF-04457845 has potential as a therapeutic candidate for ALS.

PF-04457845 is a FAAH inhibitor. FAAH degrades NATs and NAEs, and knockdown of FAAH partially attenuates motor dysfunction in SOD1^G93A^ transgenic mice (28). Based on the results of cellular screening and the putative mechanism of the hit compounds, we further investigated PF-04457845 in vivo.

### In vivo analysis of hit compounds in SOD1^G93A^ transgenic mice.

**Figure 5:**
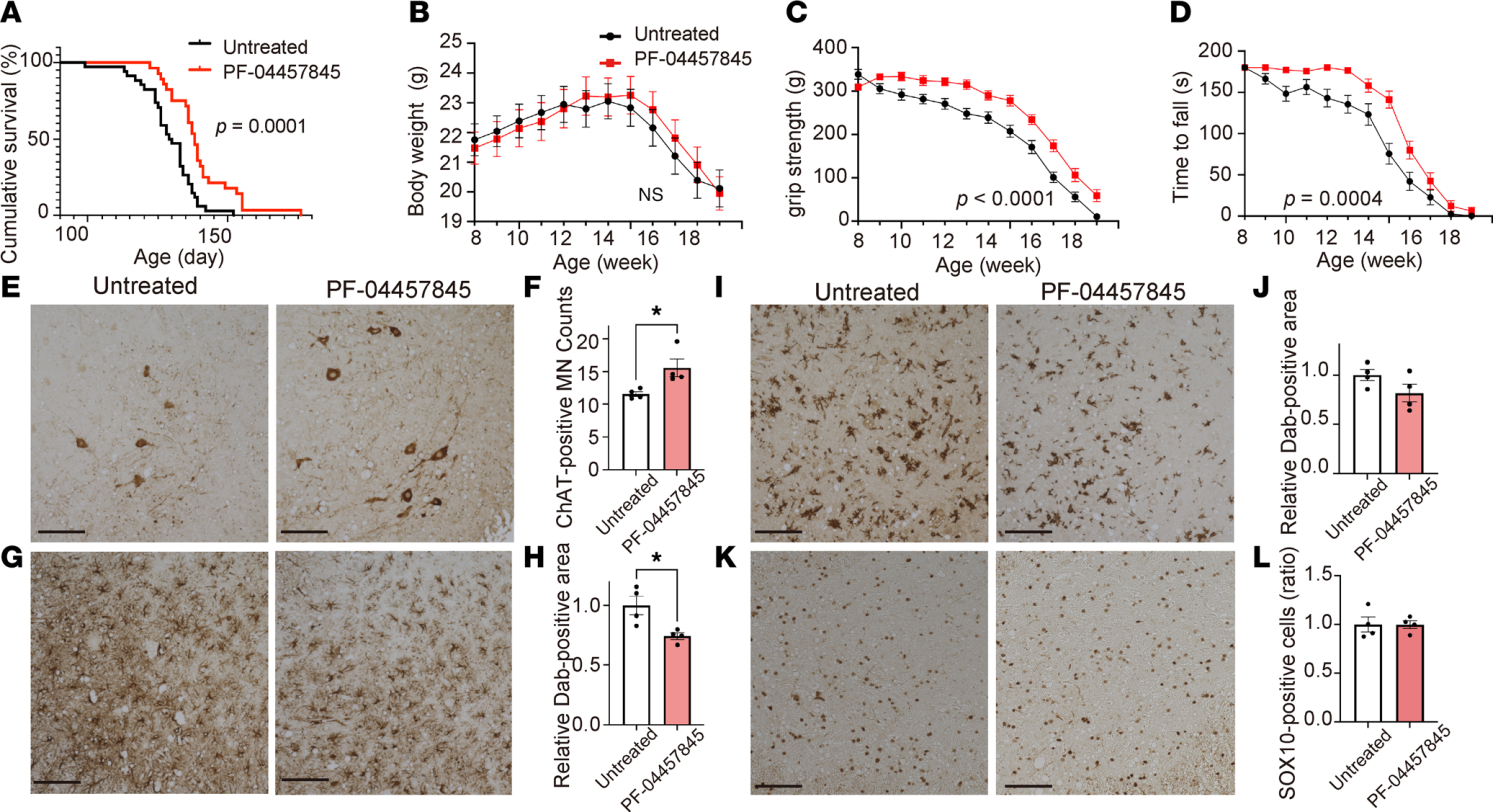
In vivo analysis of hit compounds in SOD1^G93A^ transgenic mice. (**A**) Survival of SOD1^G93A^ transgenic mice treated with PF-04457845 (~1 mg/kg/day, PF-04457845) or unmodified chow (Untreated) (*n* = 28, 34; median survival, 138, 129.5 days, respectively; *P* = 0.0001, log-rank test). PF-04457845 extended the survival of SOD1^G93A^ transgenic mice compared with untreated mice. (**B**–**D**) Body weight, grip strength, and rotarod test in SOD1^G93A^ transgenic mice treated with PF-04457845 or unmodified chow. Body weight was not significantly different, whereas grip strength (*P* < 0.0001) and rotarod test (*P* = 0.0004) were significantly improved by PF-04457845 (2-way ANOVA). To impute values after the endpoint, the final body weights were carried forward and the values of grip strength and rotarod were imputed to zero. (**E**–**L**) IHC images and quantitative analysis of immunoreactivity for choline acetyltransferase (ChAT) (**E** and **F**), glial fibrillary acidic protein (GFAP) (**G** and **H**), ionized calcium-binding adapter molecule 1 (IBA-1) (**I** and **J**), and Sry-related HMG-BOX gene 10 (SOX10) (**K** and **L**) in the ventral horn of the spinal cords of 16-week-old mice treated with PF-04457845 (*n* = 4) or unmodified chow (*n* = 4). Scale bars: 100 μm. Data are shown as mean ± SEM. Survival was analyzed using the Kaplan-Meier method and compared by the log-rank test. Body weight and motor functions were analyzed by 2-way ANOVA, and *P* values for the treatment effect are shown in the figure. Quantified pathology measures were compared using 2-sided unpaired *t* tests. *P < 0.05.

The treatment of SOD1^G93A^ transgenic mice with PF-04457845 was started at the age of 8 weeks and continued until the ethical endpoint. Body weight measurements, grip strength, and rotarod tests were repeated weekly from the age of 8 weeks to 19 weeks, which was the end stage of this model. PF-04457845 was mixed with chow at a dose of 0.001%, which corresponded to ~1 mg/kg/day and was administered to SOD1^G93A^ transgenic mice. The survival of SOD1^G93A^ transgenic mice administered PF-04457845 was extended by 8.5 days compared with that of untreated mice (Figure 5A). The administration of PF-04457845 significantly improved grip strength and rotarod test, whereas body weight was not significantly different (Figure 5, B–D). Survival and motor functional tests were also analyzed separately by the sex of the mice, yielding similar results (male mice, Supplemental Figure 10, A–D; female mice, Supplemental Figure 10, E–H). To assess possible nonspecific effects, PF-04457845 was also administered to WT C57BL/6 and B6/SJL mice. However, no significant effect of treatment was observed on body weight, grip strength, or rotarod performance, supporting the idea that its beneficial effects are disease-specific (Supplemental Figures 11 and 12).

To examine the effect of PF-04457845 on the motor neurons of SOD1^G93A^ transgenic mice, paraffin-embedded sections of lumbar spinal cord (L5) samples from 16-week-old mice were analyzed by immunohistochemistry. The number of motor neurons with immunoreactivity for choline acetyltransferase within the lumbar spinal cord was significantly preserved in the SOD1^G93A^ mice treated with PF-04457845 compared with untreated mice (Figure 5, E and F). The proliferation of astrocytes, microglia and oligodendrocytes was evaluated with anti–glial fibrillary acidic protein (anti-GFAP) antibody (Figure 5, G and H), anti–ionized calcium-binding adapter molecule 1 (anti–IBA-1) antibody (Figure 5, I and J), and anti–Sry-related HMG-BOX gene 10 (anti-SOX10) antibody (Figure 5, K and L), respectively. The proportion of astrocytes was significantly decreased by PF-04457845 treatment, although the proportions of microglia and oligodendrocytes were unchanged.

### Lipidomics analysis of the murine spinal cords.

**Figure 6:**
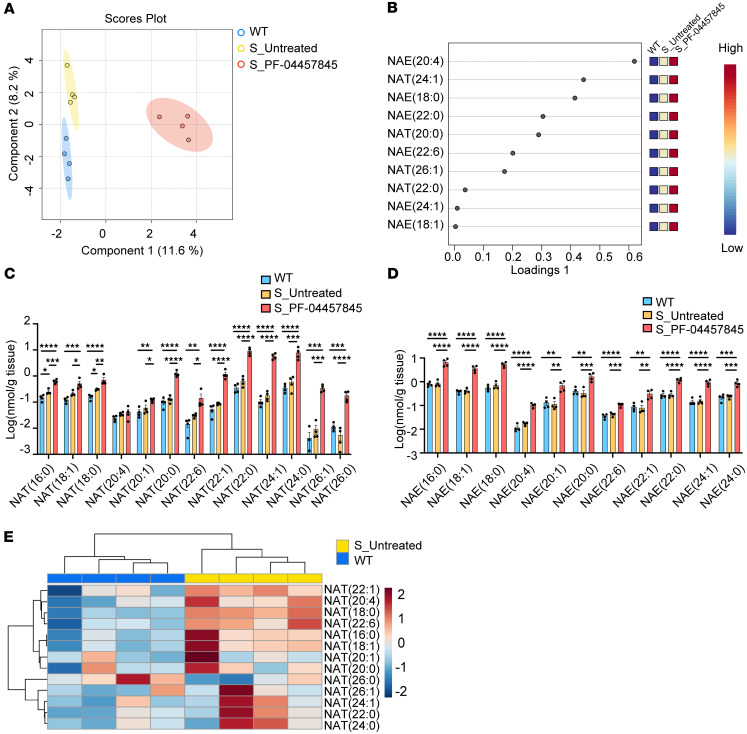
Lipidomics analysis of spinal cords of WT and SOD1^G93A^ transgenic mice with or without treatment with PF-04457845. (**A**) Sparse partial least squares discriminant analysis (sPLS-DA) plots by MetaboAnalyst 6.0 to discriminate WT mice (WT, *n* = 4), untreated SOD1^G93A^ transgenic mice (S_Untreated, *n* = 4), and SOD1^G93A^ transgenic mice treated with PF-04457845 (S_PF-04457845, *n* = 4). Loading plots of the top 10 lipids by sPLS-DA are shown in **B**. (**C**) Log transformed concentrations of *N*-acyl taurines (NAT). (**D**) Log transformed concentrations of *N*-acyl ethanolamine (NAE). (**E**) Heatmap analysis of *N*-acyl taurines comparing untreated SOD1^G93A^ transgenic mice and WT mice. Data are shown as mean ± SEM. *P < 0.05, **P < 0.01, and ***P < 0.001, ****P < 0.0001. One-way ANOVA and Tukey’s post hoc analysis were performed.

To clarify the influence of PF-04457845 on lipid metabolism, a comprehensive lipidomics analysis was conducted on the spinal cords of 3 distinct groups: wild-type mice, untreated SOD1^G93A^ transgenic mice, and SOD1^G93A^ transgenic mice treated with PF-04457845. A total of 799 lipid metabolites were identified in the process (Supplemental Table 7). Subsequent sPLS-DA analysis effectively segregated these 3 groups, underscoring that the differentiation was primarily attributed to the concentrations of NATs and NAEs, both of which are known substrates of FAAH (Figure 6, A and B). By examining the levels of specific metabolites belonging to NATs and NAEs, we found that both lipids experienced an increase after treatment with PF-04457845, demonstrating that orally administered PF-04457845 successfully upregulates NATs and NAEs in the central nervous system of the SOD1^G93A^ transgenic mice. (Figure 6, C and D). Furthermore, 3 of the 4 NAT species identified in the sera of patients with ALS — *N*-palmitoyl taurine(16:0), *N*-stearoyl taurine(18:0), and *N*-oleoyl taurine(18:1) — were detected by lipidomics. Notably, the levels of all these NATs were elevated in the spinal cord of untreated SOD1^G93A^ transgenic mice compared with WT mice, being consistent with the increase observed in patients with rapid ALS. Such changes were not observed for NAEs (Figure 6, C–E, and Supplemental Figure 13). These results indicate that elevation of neural NATs levels represents a common pathogenic mechanism shared by SOD1^G93A^ transgenic mice and patients with Rapid ALS.

### RNA and protein analysis of the spinal cord of SOD1^G93A^ transgenic mice treated with PF-04457845.

**Figure 7:**
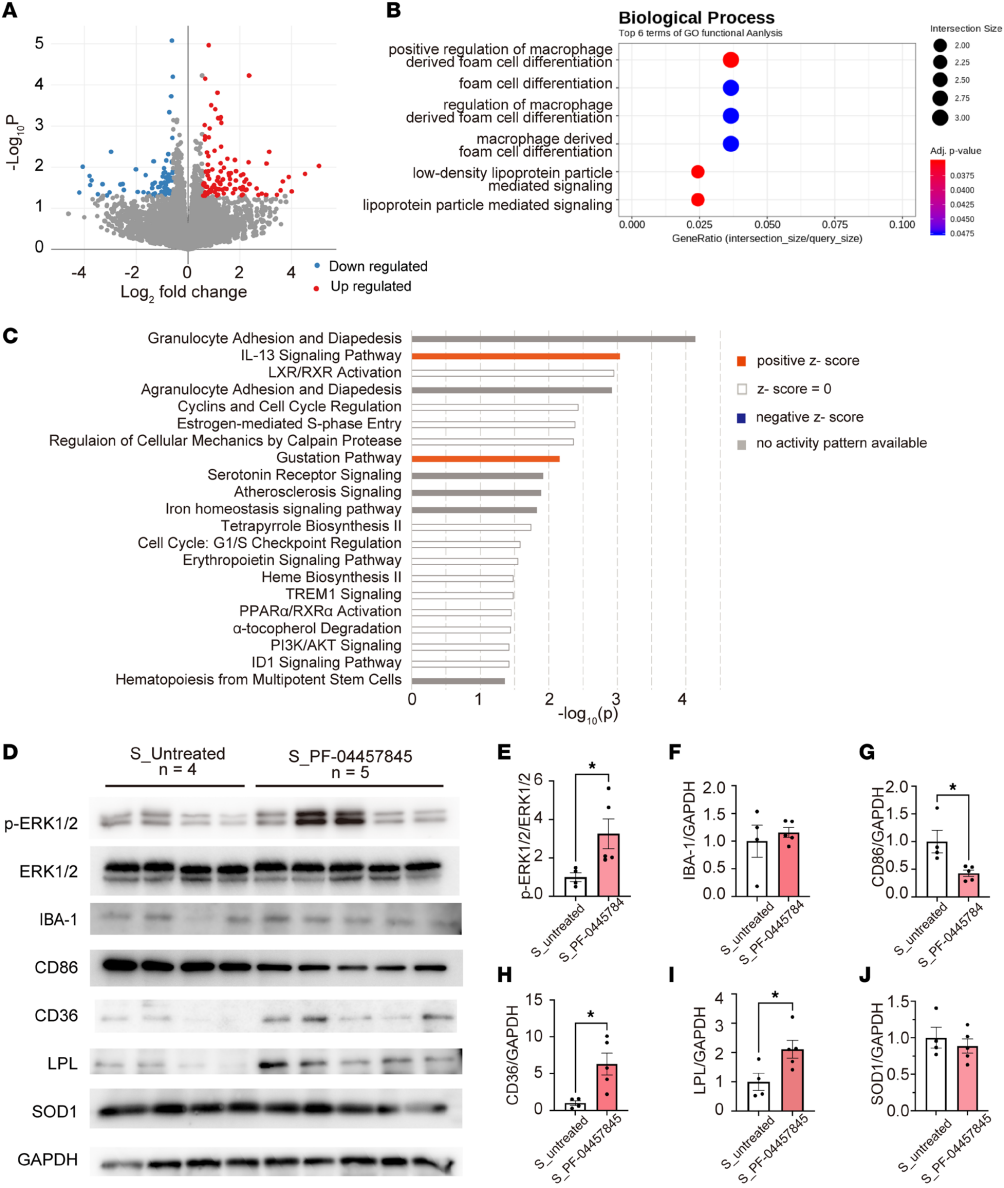
RNA-Seq analysis of the spinal cords from SOD1^G93A^ transgenic mice treated with PF-04457845. (**A**) Volcano plot of the differentially expressed genes of the bulk spinal cords between the mice treated with PF-04457845 (*n* = 4) and the untreated mice (*n* = 4). (**B** and **C**) Gene Ontology enrichment analysis for up-regulated genes (**B**) and Ingenuity Pathway Analysis (IPA) (**C**) analysis of genes with significant expression changes (fold change < 1/1.5 or > 1., *P* < 0.05) in the spinal cords of 16-week-old SOD1^G93A^ transgenic mice treated with PF-04457845 (*n* = 4) compared with untreated mice (*n* = 4). (**D**) Western blot analysis of proteins downstream of cannabinoid receptor type 2 (CB2), microglial markers, and superoxide dismutase 1 (SOD1) in the spinal cords of 16-week-old SOD1^G93A^ transgenic mice treated with PF-04457845 (S_PF-04457845, *n* = 5) and untreated SOD1^G93A^ transgenic mice (S_untreated, *n* = 4). (**E**–**J**) Quantitative analysis of phosphorylated ERK/ERK (**E**), ionized calcium-binding adapter molecule 1 (IBA-1) (**F**), CD86 (**G**), CD36 (**H**), lipoprotein lipase (LPL) (**I**), and SOD1 (**J**). The levels of IBA-1, CD86, CD36, LPL, and SOD1 were normalized to GAPDH levels. Data are shown as mean ± SEM. *P < 0.05, unpaired 2-sided *t* test.

To explore the mechanism of phenotypic amelioration in SOD1^G93A^ transgenic mice by PF-04457845, we performed RNA-Seq on their spinal cord. Enrichment analysis of the RNA-Seq data revealed significant involvement of pathways related to macrophage and microglial differentiation and lipoprotein metabolism in PF-04457845–treated mice (*n* = 4) compared with untreated mice (*n* = 4) (Figure 7, A and B, and Supplemental Table 8). Ingenuity Pathway Analysis (IPA) of the RNA-Seq data revealed activation of the IL-13 pathway in SOD1^G93A^ transgenic mice treated with PF-04457845 (Figure 7C). In line with this finding, *Cd36*, a downstream molecule of IL-13, was upregulated in the spinal cord of PF-04457845–treated mice (Supplemental Figure 14). CD36^+^ microglia regulate lipid metabolism, promote myelin debris clearance, and support antioxidant responses (29, 30). Lipoprotein lipase (LPL) is also known to be expressed in microglial subtypes such as disease-associated microglia (DAM) and proliferative region–associated microglia (PAM) (31, 32). Stimulation of CB2, an endocannabinoid receptor expressed on microglia, is reported to suppress inflammatory signaling and promote an antiinflammatory, metabolically supportive microglial state (33, 34). Therefore, we examined microglial markers and molecules downstream of CB2 receptor signaling in the mouse spinal cord by Western blotting. ERK1/2 are key kinases in the MAPK pathway and function as downstream effectors of cannabinoid receptor associated signaling. The ratio of phosphorylated ERK1/2 to total ERK1/2 was increased in PF-04457845–treated mice compared with untreated mice, suggesting activation of the CB1/CB2 signaling pathway (Figure 7, D and E) (35). The protein levels of IBA-1 were not significantly changed by PF-04457845 treatment, consistent with the histopathological findings (Figure 7, D and F). The protein levels of CD86, a proinflammatory microglial marker, were decreased, whereas those of CD36 and LPL were increased in PF-04457845–treated mice, supporting a shift toward a neuroprotective microglial phenotype (Figure 7, D and G–I). The expression of SOD1 was not affected by PF-04457845 (Figure 7, D and J).

### Transcriptome alteration in neurons by PF-04457845.

**Figure 8:**
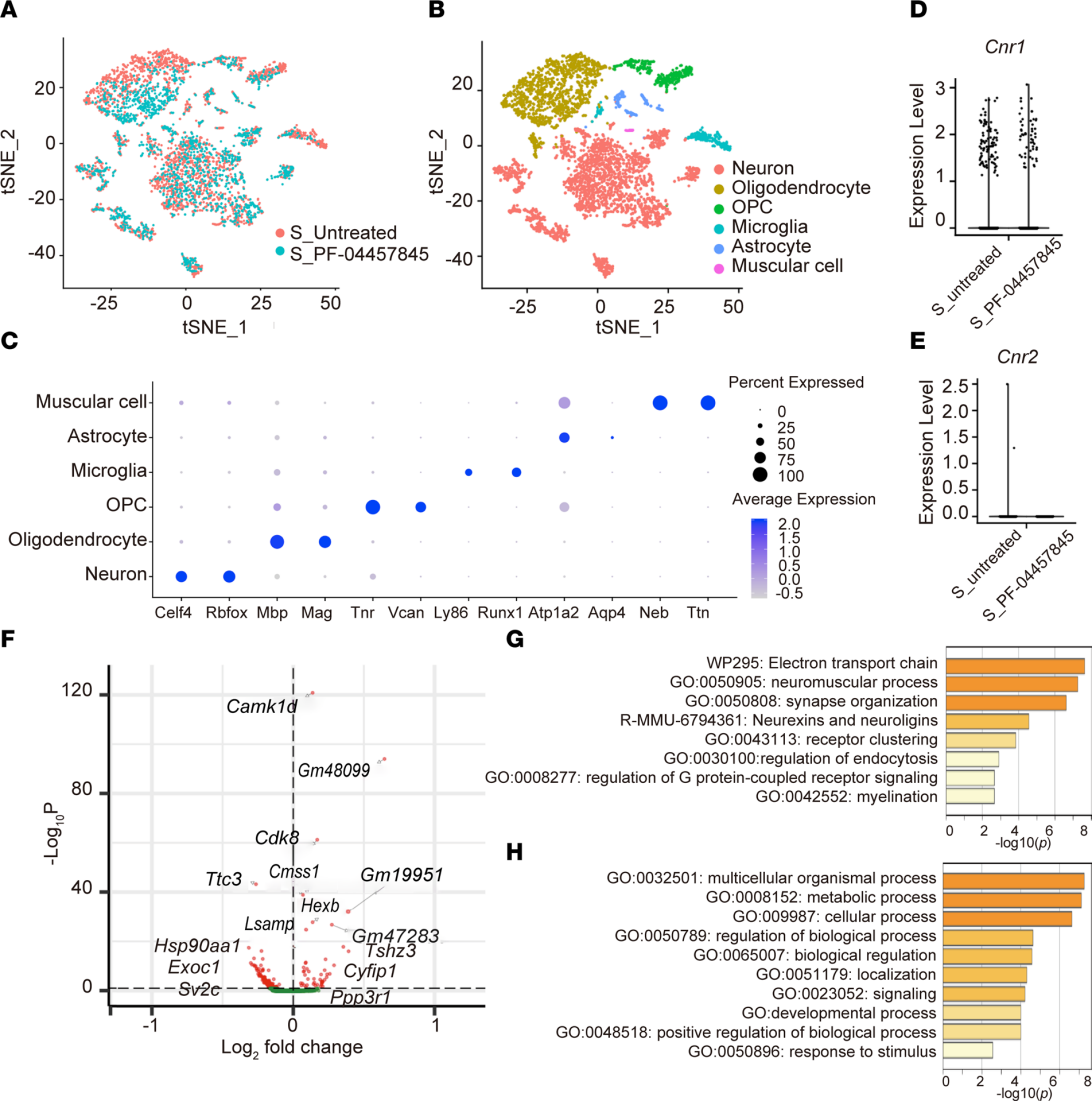
Single-nucleus RNA-Seq analysis of the spinal cords from SOD1^G93A^ transgenic mice treated with PF-04457845. We performed single-nucleus RNA-seq on the spinal cords of 16-week-old SOD1^G93A^ transgenic mice treated with PF-04457845 (S_PF-04457845) or unmodified chow (S_Untreated). (**A**) t-SNE plot color-coded by sample, with red for untreated mice and blue for PF-04457845–treated mice. (**B**) t-SNE plots of all cells sequenced showing 6 cell types. (**C**) Dot plot depicting the expression of specific markers for each cell type. (**D** and **E**) Expression levels of CB1 (**D**) and CB2 (**E**) in the neuron cluster of each sample. (**F**) Volcano plot of the differentially expressed genes in the neuron cluster from the mice treated with PF-04457845 compared with that from the untreated mice. (**G** and **H**) Gene Ontology enrichment analysis for upregulated (**G**) and downregulated (**H**) genes between the neuron clusters from the mice treated with PF-04457845 and the untreated mice.

The bulk RNA-Seq of the spinal cord primarily revealed altered signaling related to the CB2 receptor, which is mainly expressed in microglia and oligodendrocytes. Given that neurons, as well as astrocytes, express CB1 receptors, the treatment-related changes in neurons did not appear to be reflected in the RNA-Seq data. To examine the effects of treatment with PF-04457845 on neurons, we performed single-nucleus RNA-Seq (snRNA-Seq) on the spinal cords from SOD1^G93A^ transgenic mice treated with PF-04457845 or unmodified chow (Figure 8A and Supplemental Table 9). Sequencing quality was high, with > 90% of bases at Q30 in the RNA reads, a mean of 50,000 reads per cell, and > 76% of reads mapped to the genome. Unsupervised clustering identified 6 major cell types, and each cluster expressed cell type–specific cell markers (Figure 8, B and C). The cluster of neurons expressed *Cnr1*, the gene encoding the CB1 receptor; however, *Cnr2*, the CB2 receptor, was barely expressed in the neuronal population (Figure 8, D and E). The administration of PF-04457845 upregulated the expression of genes including *Tshz3*, *Ppp3r1*, and *Cyfip1* in neurons (Figure 8F). Enrichment analysis revealed that PF-04457845 activates synapse plasticity and neuronal development, although no specific pathway was downregulated with the treatment (Figure 8, G and H). In addition, although the abundance of *Cnr1*^+^ neurons was not significantly altered, pathway analysis revealed treatment-associated transcriptional changes in this neuronal subset (Supplemental Figure 15). In oligodendrocytes, pathway analysis revealed treatment-associated changes related to myelination, oligodendrocyte differentiation, and cytoskeletal organization, suggesting transcriptional remodeling without a major change in oligodendrocyte abundance (Figure 5, K and L, and Supplemental Figure 16). In microglia, PF-04457845 treatment was associated with downregulation of pathways related to cytokine production, and cytoskeletal/cell migration programs, together with upregulation of pathways related to synapse organization and trans-synaptic signaling (Supplemental Figure 17).

## Discussion

In the present study, metabolome and lipid mediator analysis of patients’ sera identified metabolic pathways associated with the progression rates of sporadic ALS. Investigation of individual metabolites showed that elevated serum levels of NATs were correlated with longitudinal changes in the ALSFRS-R and associated with shorter survival. Based on these clinical findings, we performed an in vitro screening of compounds that target the identified metabolic pathways. Among the hit compounds in the cellular assay using NSC-34 cells and iPSC-MN derived from patients with ALS, PF-04457845, an FAAH inhibitor which upregulates NATs and NAEs, ameliorated motor neuron degeneration in SOD1^G93A^ transgenic mice, presumably through the regulation of microglial and neuronal function. These findings indicate the elevation of NATs is a progression-related biomarker of sporadic ALS, and manipulation of NAT metabolism, especially inhibition of FAAH, can be a therapeutic strategy.

Based on the data analysis of sporadic ALS patient serum metabolomics, we selected the following 5 key metabolic pathways that showed differences between rapidly and slowly progressive groups: NAT, purine/xanthine, polyamine/arginine, bile acid, and acylcarnitine metabolism. Previous studies on metabolomics in ALS analyzed the plasma or cerebrospinal fluid (CSF) of patients and healthy individuals, although the results vary due to differences in samples and metabolomic methodologies (36–44). The present study partially reproduced the results of such previous studies. For instance, we identified altered serum levels of adenine, xanthosine, and xanthine in patients with ALS. Alterations in purine/xanthine pathways have also been reported in previous studies (40, 42), suggesting that reactive oxygen species (ROS) generated by xanthine oxidase are related to ALS etiology. Our study also demonstrated alterations in the levels of acylcarnitine, a metabolite involved in β-oxidation of fatty acids in mitochondria, which has been reported in previous metabolomic studies (43, 44). Another finding of the present study is altered polyamine/arginine metabolism in sporadic patients with ALS. Our results show that serum levels of spermidine and [*N*(1) + *N*(8)]-acetylspermidine were increased in the rapidly progressive ALS group. A previous report exploring human plasma and SOD1^G93A^ transgenic mice plasma, cerebral cortex, and muscle tissue by metabolomics identified alterations in polyamine metabolism as common metabolic phenotypes of human ALS and SOD1^G93A^ transgenic mice (41). Although sphingolipid metabolism was not prioritized among the candidate pathways identified in our metabolomic analyses, sphingosine-related alterations were also observed in the rapid-versus-slow comparison. This is broadly consistent with prior reports implicating sphingolipid dysregulation in ALS, including *SPTLC1*-associated pathogenesis (45, 46).

The major discovery of the present study is that NATs, lipid mediators that are included in the expanded ECS (35, 47), are upregulated in sporadic patients with ALS with rapid progression. NATs are chiefly produced by conjugation of taurine with fatty acyl chains by bile acid-CoA: amino acid N-acyltransferase (BAAT) in the liver, whereas the biosynthetic pathway of NATs in the central nervous system remains unclear (48). The serum levels of NATs were increased in the rapidly progressive ALS group, being consistent with the result of lipidomics analysis on the spinal cord of SOD1^G93A^ transgenic mice. Furthermore, the serum levels of NATs were closely correlated with the longitudinal decrease in the ALSFRS-R rating and survival in the separate 2 cohorts, while major endocannabinoids, AEA and 2-AG, were unchanged in patients with ALS. Although functions of NATs in the central nervous system have yet to be elucidated, our results suggest that NATs are aberrantly regulated in patients and the mouse model of ALS and associated with disease progression in sporadic ALS.

In our cellular experiments, neuroprotective effects of PF-04457845, an inhibitor of FAAH, and *N*-stearoyl taurine, one of NAT species, are stronger than those of AEA or 2-AG, and the effectiveness of PF-04457845 was reproducible in iPS-MN derived from patients with ALS. Moreover, pharmacological inhibition of FAAH led to a substantial increase in the levels of both NATs and NAEs in the spinal cord, resulting in phenotypic amelioration of the mutant SOD1 mice. Collectively, targeting the expanded ECS, including NATs and NAEs, appears to be a common therapeutic approach for both patients and the animal model of ALS. Our findings also suggest that the elevated levels of NATs in rapidly progressive ALS are indicative of compensatory but insufficient metabolic responses, and that further upregulation of this pathway may mitigate disease progression. PF-04457845 is known to increase the concentration of endocannabinoids in tissues, including those in the central nervous system (49), and has been examined in a phase 2 clinical trial for pain associated with osteoarthritis, which demonstrated the safety and tolerability of this compound in humans (50). Our results, thus, suggest that PF-04457845 is a candidate for disease-modifying therapy for ALS. Given that NAT synthesis is also linked to hepatic enzyme BAAT (48), regulation of such enzyme may be an alternative potential therapeutic strategy for ALS.

Major molecules of the ECS act through endocannabinoid receptors CB1 and CB2. For instance, NAEs including AEA bind to CB1 and CB2 as well as to transient receptor potential cation channel subfamily V member 1 (TRPV1), while NATs are known as ligands for TRPV1 and TRPV4. In previous studies, activation of the CB1 and CB2 pathways demonstrated potential benefits in SOD1^G93A^ mice (28, 51). The bulk RNA-Seq data and Western blot on the spinal cords from treated mice revealed activation of the IL-13 and CB2 signaling pathways, both of which are known to promote a neuroprotective microglial state (35). It is also known that the activation of TRPV1 regulates microglial lipid metabolism and inflammation (52). The effect of PF-04457845 in our study is, at least partially, attributable to microglia adopting a neuroprotective phenotype, characterized by enhanced antiinflammatory signaling and lipid metabolic support, as indicated by the increased protein levels of CD36 and LPL, which are specifically expressed in microglia. In addition, our study also demonstrated the benefits of PF-04457845 in NSC-34 cells expressing pathogenic TDP-43 or SOD1 protein and iPS-MN derived from patients with ALS, suggesting the effect of this compound on neurons. In support of this view, the results of snRNA-Seq indicated that *Tshz3*, *Cyfip1*, and *Ppp3r1* are upregulated in neurons treated with PF-04457845 in vivo. Tshz3 is essential for cerebral cortical projection neuron development and has been implicated in the pathogenesis of autism spectrum disorder (ASD) (53). CYFIP1 is also related to ASD and schizophrenia and is known to play an important role in dendritic spine maturation, synaptic activities, and plasticity and acts with CB1 (54–58). Ppp3r1 is a calcineurin subunit that is required for neuronal survival and neurite outgrowth of mesencephalic dopaminergic neurons by glial cell line-derived neurotrophic factor (59). Variants in *PPP3R1* are associated with the accelerated progression of Alzheimer’s disease (60). Altogether, in addition to the microglial mechanism mentioned above, PF-04457845 appears to have direct protective effects on neurons via CB1-mediated pathways.

The present study has several limitations. First, the sample size of the clinical study was small, even though we analyzed 2 separately recruited clinical cohorts. Therefore, larger validation studies are needed. Second, we utilized 2 murine cellular models for in vitro screening and SOD1^G93A^ transgenic mice for in vivo analysis of motor neurons and glial cells. These models do not necessarily reflect the pathogenesis of sporadic ALS, though our study demonstrated the common elevation of NATs in rapid progressive patients with ALS and the murine model. Finally, the efficacy of the FAAH inhibitor PF-04457845 seems superior to similar approaches in the literature, but the pharmacological basis for the difference remains unclear.

### Conclusion

Our study suggests that the dysregulation of NATs may serve as a potential biomarker for ALS progression, representing a common therapeutic target in the pathophysiology of both human ALS and an animal model. Furthermore, the FAAH inhibitor PF-04457845, which up-regulates NATs and NAEs in murine spinal cords, emerges as a promising disease-modifying therapy for ALS.

## Methods

### Sex as a biological variable

The clinical study included both male and female participants. Sex-stratified analyses of NAT levels revealed similar trends across both sexes. When PF-04457845 treatment was applied to SOD1^G93A^ transgenic mice, consistent improvements in motor function and survival were observed in both male and female mice. Pathological analyses were performed using male SOD1^G93A^ transgenic mice to ensure consistency and sensitivity, as males exhibit a more severe and uniform ALS phenotype (61).

### Participants

Patients who were clinically diagnosed with the revised El Escorial criteria (62) of definite, probable, or possible ALS were consecutively recruited separately for a discovery cohort and a replication cohort. The principal inclusion criteria were no family history and disease duration of ≤ 2 years at the time of enrollment. Patients who had severe complications such as malignancy, heart failure, or renal failure and those with Mini-Mental State Examination (MMSE) score of ≤ 23 were excluded from this study. For the discovery cohort, a total of 26 patients with ALS and 10 healthy controls were analyzed; for the replication cohort, a total of 55 patients with ALS and 25 healthy controls were analyzed. Patients with sporadic ALS were assessed during hospitalization at the initial evaluation and follow up evaluations at an outpatient clinic or by telephone were conducted every 6 months in the discovery cohort. In the replicate cohort, the second evaluation was performed 3–11 months after the initial assessment. Age- and sex-matched healthy controls were also recruited during the same period as the patients with ALS. All study patients were Japanese and observed at the Nagoya University Hospital, Nagoya, Japan, between May 2013 and June 2025.

### Clinical evaluation and sample collection

Disease onset was defined as the time point when the patient felt weakness of any body part. Patients with ALS were classified according to onset type and were sorted into either the limb-onset type group or the bulbar-onset type group, according to the site where they first felt weakness. Disease severity was assessed with the Japanese version of the revised ALS Functional Rating Scale (ALSFRS-R), a validated questionnaire-based functional rating scale for ALS (63). The introduction of TPPV or death of the patient was defined as the primary endpoint.

At the first evaluation, venous blood samples were collected from patients with ALS in the supine position after more than 12 hours of fasting and just after waking up during hospitalization. At the outpatient clinic, venous blood samples of healthy individuals in the sitting or supine position were collected after more than 12 hours of fasting. Serum samples were centrifuged at 1, 400*g*) for 10 minutes and stored at –80°C until processing at Metabolon Inc. and Shimadzu Techno-Research Inc.

### Metabolomics analysis

Untargeted metabolomics profiling of serum samples was performed using ultrahigh-performance liquid chromatography-tandem mass spectrometry (UPLC-MS/MS) by Metabolon Inc. To remove protein, dissociate small molecules bound to protein or trapped in the precipitated protein matrix and to recover chemically diverse metabolites, proteins were precipitated with methanol. The resulting extract was divided for analysis by 2 separate reversed-phase (RP)/UPLC-MS/MS methods with positive ion mode electrospray ionization (ESI), for analysis by RP/UPLC-MS/MS with negative ion mode ESI, and for analysis by HILIC/UPLC-MS/MS with negative ion mode ESI. Compounds were identified by comparison to the library entries of purified standards or recurrent unknown entities, and relative levels were quantitated. Missing values were replaced by the minimal value determined for that metabolite in the entire cohort, per Metabolon protocols.

### Lipid mediator analysis

To validate the alterations in NATs observed in the discovery cohort, lipid mediators in serum samples from the replication cohort were measured and compared using LC-MS/MS by Shimadzu Techno-Research Inc. Sample preparation was performed according to the Shimadzu lipid mediator method package. Missing values were imputed with the minimum detected value for each metabolite across the entire cohort, and metabolites for which more than 50% of the values were below the limit of detection were excluded from the analysis.

### Metabolomics data analysis

Volcano plots were created and 1-way ANOVA, heatmap analysis, and enrichment analysis were performed using MetaboAnalyst 6.0 (64). For multivariate analysis, sPLS-DA was performed to discriminate between patients with rapid ALS, patients with slow ALS and healthy individuals using MetaboAnalyst 6.0. Loading plots indicated the importance of metabolites for sPLS-DA. Adding to this multivariate analysis, random forest (RF) classification in R software was applied to determine whether the differences in the identified metabolites effectively separated the 3 groups. The mean decrease in accuracy, which represents variable importance, was calculated.

### ALS cellular models, cell culture, and transfection

Mouse NSC-34 motor neuron-like cells (provided by N.R. Cashman, University of British Columbia, Vancouver, British Columbia, Canada) were cultured in a humidified atmosphere of 95% air/5% CO_2_ in a 37°C incubator. NSC-34 cells were maintained in Dulbecco’s Modified Eagle’s Medium (DMEM) containing 10% (v/v) fetal bovine serum (Life Technologies) with 1% penicillin/streptomycin (Wako). The plasmids were transfected by using OPTI-MEM (Gibco) and Lipofectamine 2000 (Invitrogen) according to the manufacturer’s instructions. NSC-34 cells were differentiated in DMEM for 48 hours. We prepared the mutant TDP-43^A315T^ and the mutant SOD1^G93A^ vectors as previously described (65, 66). As for TDP-43, site-specific mutagenesis was performed as described previously using the following primers: F: acgttcagcattaatccagccatgatg R: accaaagttcatcccaccacccatattactac.

### hiPSC culture and differentiation in vitro

The experiments were performed as described previously (67–69). Briefly, hiPSCs derived from 3 patients with ALS and a healthy individual (HPS0736, 0760, 0896 and 0063 obtained from the RIKEN BioResource Research Center) (70, 71) were maintained on feeder cells with human embryonic stem cell medium. For differentiation, hiPSC colonies were detached and embryoid bodies were generated by human embryoid body medium with LDN, SB, CHIR, retinoic acid, and purmorphamine for 14 days. Embryoid bodies were dissociated into single cells using TrypLE Select and were plated on dishes coated with growth factor reduced Matrigel at a density of 2 × 10^5^ cells/cm^2^. Motor neurons were cultured in motor neuron medium consisting of B27 supplement, CultureOne supplement, nonessential amino acid, retinoic acid, purmorphamine, cyclic AMP, recombinant brain-derived neurotrophic factor, recombinant glial cell line derived neurotrophic factor, recombinant human insulin-like growth factor-1, and L-ascorbic acid for up to 3 weeks. Half of the medium was changed every 2–3 days.

PF-04457845 was administered at 0 nM, 20 nM, and 40 nM to motor neurons cultured for 2 weeks. The number of cell cluster and neurite length were evaluated by IncuCyteSX5 live-cell imaging system (Essen Bioscience) on day 7 after drug administration. Cell clusters and neurite length were assessed using the NeuroTrack analysis algorithm. LDH release was also assessed as the drug efficacy.

### Chemical compounds

PF-04457845 was supplied by Mitsubishi-Tanabe Pharma, and the other compounds were commercially purchased from Cayman Chemical (arvanil, N-oleoyl taurine, N-stearoyl taurine, N-palmitoyl taurine); Kanto Chemical (xanthine); MedChemExpress (eflornithine); Focus Bioscience (Mitoquazone); Sigma-Aldrich (allantoin, AEA, clofibrate, febuxostat, inosine, metformin, MDL72527, oleic acid, riboflavin, S-adenosyl-L-methionine, Spermidine, Spermine, and 2-arachidonoyl glycerol); TCI (agmatine, glicocholic acid, glycoursodeoxycholic acid, riluzole, theobromine, theophiline, trimetazidine, and xanthosine), Matrix Scientific (edaravone), or Wako (arginine and ornithine).

### Cell viability and toxicity assay

Cell viability assays were performed using a WST-8 kit (Dojindo) according to the manufacturer’s instructions. Transfected and differentiated NSC-34 cells were cultured in 96-well plates, and chemical compounds were administered. Twenty-four hours after treatment with the selected chemicals, cells were incubated with the WST-8 substrate for 30–60 minutes, after which the absorbance of the wells was measured at 450 nm using a plate reader (EnSpire, PerkinElmer). Toxicity assays were performed using the Cytotoxicity Detection Kit PLUS (Roche Diagnostics). Twenty-four hours after treating the cells, the medium was incubated with the substrate for 15 minutes and spectrophotometrically assayed at 492 nm using a plate reader.

### Preclinical murine model of ALS

Transgenic mice overexpressing the human SOD1 gene carrying the G93A mutation were purchased from the Jackson Laboratory and maintained as hemizygotes by mating transgenic males with B6/SJL F1 females. The SOD1^G93A^ transgenic mice were randomly allocated to PF-04457845–treated (*n* = 28; male *n* = 13, female *n* = 15), or untreated groups (*n* = 34; male *n* = 17, female *n* = 17), according to the guidelines for preclinical animal research in ALS/MND (72). For oral administration of PF-04457845, PF-04457845 was mixed with powdered rodent chow at a concentration of 0.001%, which is consistent with the dose of 1 mg/kg/day. All mice were placed under a 12-hour light/12-hour dark cycle in a temperature- and humidity-controlled facility and had unlimited access to food and water. PF-04457845 (1 mg/kg/day) was administered from week 8 until the ethical endpoint, which was defined as loss of the ability to right itself after being placed on its side or inability to ambulate from a given location. The endpoint was evaluated daily. PF-04457845 was also administered to wild-type C57BL/6 and B6/SJL mice to assess possible nonspecific effects on body weight and motor performance. In C57BL/6 mice, each group included 10 animals (male, *n* = 5; female, *n* = 5), whereas in B6/SJL mice, each group included 9 animals (male, *n* = 5; female, *n* = 4).

### Behavioral analysis

All behavioral tests were performed weekly, and the data were prospectively analyzed. The animal rotarod performances were assessed weekly using an Economex Rotarod during the light phase of the 12-hour light/12-hour dark cycle as described previously (73) (Ugo Basile). We performed 3 trials and recorded the longest duration on the rod for every mouse in a randomized order. We stopped the timer when the mouse fell from the rod or after a maximum of 180 seconds. The grip strength was measured with a Grip Strength Meter (MK-380M, Muromachi Kikai). Behavioral analysis was performed by 2 evaluators blind to the treatment. To impute values of mice after death, the final body weights of all mice were carried forward when calculating group mean values as described in a previous report (73). The values of grip strength and rotarod performance after death were imputed to zero.

### Animal sample collection

Male mice were deeply anesthetized, and the entire spinal cord was dissected and snap-frozen in powdered CO_2_ in acetone for Western blot and RNA-seq and by liquid nitrogen for lipidome analysis. The mice were sacrificed at the age of 16 weeks. Mouse tissues were dissected, postfixed with 10% phosphate-buffered formalin and paraffin embedded for histological analysis.

### Immunoblotting

Mouse tissues were lysed with RIPA buffer (50 mM 1M Tris [pH 7.4], 150 mM NaCl, 1% Triton X-100, 1% deoxycholate, 0.1% SDS) using a dounce homogenizer for mouse tissue, sonicated, and centrifuged at 20,000*g* for 15 minutes at 4°C. The supernatant was kept as the RIPA soluble fraction, and equal amounts of protein were separated on 5%–20% SDS-PAGE gels (Wako) and transferred to Hybond-P membranes (GE Healthcare). The following primary antibodies and dilutions were utilized: SOD1 (ab51254, 1:1,000; Abcam), IBA-1 (016-20001, 1:1,000; Wako), CD36 (ab252923, 1:1,000; Abcam), CD86 (1858-1, 1:1,000; Epitomics), LPL (GTX101125, 1:1,000; GeneTex), p44/42 MAPK (9102, 1:5,000; CST), and phosphorylated p44/42 MAPK (5726, 1:1,000; CST). Primary antibodies bound to the proteins were probed with a 1:5,000 dilution of horseradish peroxidase–conjugated secondary antibodies, and the bands were detected using an immunoreaction enhancing solution (Can Get Signal; Toyobo) and enhanced chemiluminescence (ECL Prime; GE Healthcare). Chemiluminescence signals were digitized using a LAS-3000 imaging system (Fujifilm). The signal intensities of independent blots were quantified using ImageJ (NIH). Membranes were reprobed with an anti-GAPDH antibody (MAB374, 1:5000; Millipore) for normalization.

### Histology and IHC

The lumbar spinal cords were collected from mice. The samples were embedded in paraffin, and 3 μm sections were prepared. Sections designated for anti-ChAT, GFAP, and IBA-1, and SOX10 staining were boiled in 10 mM citrate buffer for 15 minutes for antigen retrieval, incubated overnight with the respective primary antibodies, and then incubated with a secondary antibody labeled with a polymer as part of the Envision+ system containing horseradish peroxidase (Dako Cytomation). The following primary antibodies and dilutions were used to stain mouse tissues: ChAT (AB144P, 1:100; Millipore), GFAP (ab53554, 1:1,000; Abcam), IBA-1 (013-27691, 1:1,000; Wako) and SOX10 (sc-365692, 1:500; Santa Cruz Biotechnology).

ChAT-immunoreactive neurons in the ventral horn of the lumbar spinal cord were counted in every fifth section from the 50 consecutive sections by using BZ-X810 (Keyence), and the mean total number of ChAT-immunoreactive neurons was compared between treatment groups. ChAT-immunoreactive neuronal area and GFAP^+^ and IBA-1^+^ areas were quantified using NIH ImageJ software, and SOX10-positive cells were counted.

### Lipidomics analysis of murine spinal cords

Frozen tissues (about 10 mg) were homogenized with a probe sonicator in methanol/water = 2/0.7 (v/v), and lipids were extracted using the method of Bligh and Dyer with internal standards. The organic (lower) phase was transferred to a clean vial and dried under a nitrogen stream. The lipids were resolubilized in methanol/isopropanol/chloroform = 45/45/10 (v/v/v) and stored at –80°C. A portion of the extracted lipids was injected into an ultrahigh-performance liquid chromatography–electrospray ionization–tandem mass spectrometry system (LC-ESI–LC-MS/MS). The quantification of FFA was carried out as described previously (74). For the quantification of Sulfatide, S1P, NAT, NAEP, LPA, LPG, LPI, LPS, PA, PS, and PT, LC separation was performed on an ACQUITY Premier BEH C18 column (1.7 μm, 2.1 × 50 mm; Waters). Mobile phase A was H_2_O/methanol = 95/5 (v/v%), mobile phase B was isopropanol/methanol = 63/37 (v/v%), and mobile phase C was H_2_O/methanol/28% NH_4_OH = 93/5/2 (v/v/v%). The LC method consisted of a linear gradient from A/C = 95/5 (v/v%) to B/C = 95/5 (v/v%) over 15 minutes, B/C = 95/5 (v/v%) for 8 minutes, and equilibration with A/C = 95/5 (v/v%) for 5 minutes (28 minutes total run time). The flow rate was 0.3 mL/min, and the column temperature was 25°C. For the quantification of bis(monoacylglycero)phosphate (BMP), an isocratic LC separation was performed with methanol containing 10 mM ammonium formate on a COSMOCORE 2.6C18 column (2.1 × 100 mm; Nacalai Tesque) coupled to an ACQUITY UPLC BEH C18 VanGuard Precolumn (1.7 μm, 2.1 × 5 mm; Waters). The flow rate was 0.3 mL/min, and the column temperature was 55°C. For the quantification of other lipid classes, LC separation was performed on an ACQUITY UPLC BEH C18 column (1.7 μm, 2.1 × 100 mm; Waters) coupled to an ACQUITY UPLC BEH C18 VanGuard Precolumn (1.7 μm, 2.1 × 5 mm; Waters). Mobile phase A was acetonitrile/water = 60/40 (v/v%) containing 10 mM ammonium formate and 0.1% (v/v) formic acid, and mobile phase B was isopropanol/acetonitrile = 90/10 (v/v%) containing 10 mM ammonium formate and 0.1% (v/v) formic acid. The LC gradient consisted of 20% B for 2 minutes, a linear gradient to 60% B over 4 minutes, a linear gradient to 100% B over 16 minutes, and equilibration with 20% B for 5 minutes (27 minutes total run time). The flow rate was 0.3 mL/min, and the column temperature was 55°C. Multiple reaction monitoring (MRM) was performed using a Xevo TQ-S micro triple quadrupole mass spectrometry system (Waters) equipped with an ESI source. The ESI capillary voltage was set at 1.0 kV, and the sampling cone was set at 30 V. The source temperature was 150°C, the desolvation temperature was 500°C, and the desolvation gas flow was 1,000 L/h. The cone gas flow was 50 L/h.

### Lipidomics data analysis

Detailed methods are provided in the Supplemental Methods.

### RNA-Seq of murine spinal cords

Detailed methods are provided in the Supplemental Methods.

### RNA-Seq data analysis

Detailed methods are provided in the Supplemental Methods.

### Single-nucleus RNA-Seq of murine spinal cords

#### Nuclei isolation.

Detailed methods are provided in the Supplemental Methods.

#### Single-nucleus RNA sequencing.

The nuclei per sample were run on the 10× Chromium Single cell 3′ gene expression v3.1 platform. Sequencing libraries were constructed according to the manufacturer’s instructions, and cDNA samples were run on an Agilent Bioanalyzer using the High Sensitivity DNA Chip as quality control and determination of cDNA concentrations. The samples were run on an Illumina HiSeq2500 at read 1 = 28 bp and read 2 = 90 bp, with a minimum depth of 20,000 reads per nucleus. For alignment, introns and exons were included in the reference genome (mm10) using the CellRanger v.6.1.2 pipeline (10X Genomics). Sequencing data were analyzed using the R package Seurat Version 4.1.3. The gene barcode matrices for each sample were imported into R using the Read10X function.

#### Quality check analysis.

All 10 × Genomics Chromium runs for each sample were filtered for nuclei with less than 1% contamination of mitochondrial genes, with 200–2,000 genes per cell, and genes with a count of 1 in at least 3 cells were retained. A total of 4,869 nuclei passed quality control filtering and proceeded to analysis. UMI counts were then normalized in Seurat 3.0, and the top 2000 highly variable genes were identified using the FindVariableFeatures function with variance stabilization transformation (VST).

#### Clustering and data analysis.

Principal component analysis (PCA) was performed using the top 2,000 variable genes prior to clustering. To visualize profiles in 2-dimensional space, t-distributed stochastic neighbor embedding (t-SNE) was performed with the top 25 principal components based on ElbowPlot. Clustering was performed using the FindNeighbors and FindClusters functions in the Seurat R package, and the resolution was 0.15. To identify each cell type, “Celf4” and “Rbfox” were used as specific cell markers for neurons, “Mbp” and “Mag” for oligodendrocytes, “Tnr” and “Vcan” for oligodendrocyte precursor cells (OPCs), “Ly86” and “Runx1” for microglia, “Alp1a2” and “Aqp4” for astrocytes, and “Neb” and “Ttn” for muscular cells. These markers were used to assign cell-type annotations manually for each cell cluster.

### Single-nucleus RNA-seq data analysis

DEGs between PF-04457845–treated and untreated groups were identified using the FindMarkers function of the Seurat package in R, using the Wilcoxon rank-sum test. Average log_2_ (fold change) of gene expression, the percentage of cells expressing the genes in each group (pct.1 and pct.2), *P* value, and adjusted *P* value were generated. For the neuronal analysis, DEG lists were produced by filtering all genes for log_2_-fold changes > 0.1 and adjusted *P* < 0.05. For oligodendrocytes, genes with adjusted *P* < 0.05 were extracted and then separated into upregulated and downregulated genes according to the direction of fold change. For microglia and cannabinoid receptor 1^+^ (CB1^+^) neurons, genes with *P* < 0.05 were used for downstream pathway analysis. Ontology terms with *P* < 0.01, a minimum count of 3, and an enrichment factor > 1.5 were extracted. DEGs upregulated in PF-04457845–treated mice compared with untreated mice were analyzed with the Metascape portal (www.metascape.org) (75).

### Generative artificial intelligence

ChatGPT (GPT-5.4; OpenAI) was used solely for correction of typographical errors and minor grammatical issues. All revisions were reviewed and approved by the authors. The use of this AI tool occurred on April 6, 2026.

### Statistics

We analyzed the data by using unpaired 2-tailed *t* tests and χ^2^ tests for comparisons of 2 groups and 1-way ANOVA for multiple comparisons. For multiple comparisons, we utilized Tukey’s test for comparing metabolites among patients with rapid ALS, patients with slow ALS, and healthy individuals and Dunnett’s test was used to compare each concentration of chemical compounds with baseline in ALS cell models. Data from iPSC-derived motor neurons were analyzed using a mixed-effects model with treatment as a fixed effect and iPSC line as a random effect, followed by Dunnett’s multiple-comparisons test. ALSFRS-R slope values were transformed using the Yeo-Johnson transformation. A Spearman rank correlation test was performed to analyze the correlation between ALSFRS-R slope and serum metabolite levels. In addition, survival was analyzed using Kaplan-Meier and log-rank tests for comparison of patients with rapidly progressive ALS and patients with slowly progressive ALS. The same methods were also used to compare untreated and PF-04457845–treated SOD1^G93A^ transgenic mice. Body weight, grip strength, and rotarod test in mice were analyzed using 2-way ANOVA. We considered *P* < 0.05 to indicate statistical significance, and a correlation coefficient value (*r*) of 0.40–0.59 is considered moderate, while 0.60–0.79 is considered strong in Spearman’s rank correlation coefficient. For experiments using mice, the number of animals is stated in the figure legends. All statistical analyses were performed using R (version 4.5.2) and GraphPad Prism 10.

### Study approval

The clinical part of this study was conducted according to the Declaration of Helsinki, the Ethical Guidelines for Medical and Health Research Involving Human Subjects endorsed by the Japanese government. It was approved by the Ethics Review Committee of Nagoya University Graduate School of Medicine (nos. 2013-0035 and 2015-0041), and all participants gave written informed consent before participation.

All animal experiments were performed in accordance with the *Guide for the Care and Use of Laboratory Animals* (National Academies Press, 2011), ARRIVE guideline, and under the approval of the Nagoya University Animal Experiment Committee (No. 29170).

### Data availability

The metabolomics source data were deposited into Mendeley (doi:10.17632/f8gtkwfs6s.1). The snRNA-Seq and bulk RNA-Seq raw data files have been deposited into the NCBI Gene Expression Omnibus (GEO) under accession no. GSE242942 and are publicly available at https://www.ncbi.nlm.nih.gov/geo/query/acc.cgi?acc=GSE242942

Values for all data points in graphs are reported in the Supporting Data Values file.

## Author contributions

Conceptualization was contributed by DI, MI, YI, AH, and MK. Methodology was contributed by DI, MI, YI, AH, and MK. Clinical investigation was contributed by DI, AH, S Yamada, YK, and SK. Molecular experiments were contributed by DI, MI, SN, and S Yokoi. Lipidomics was contributed by HCLO and TY. Single-nucleus RNA-Seq was contributed by MI, TA, and KH. Scientific discussions were contributed by KO, TS, YT, MN, YO, JN, GS, ST, and KT. Supervision was contributed by MI, YI, and MK. Writing of the original draft was contributed by DI. Review and editing were contributed by MI, YI, and MK.

## Conflict of interest

This study was partially supported by Mitsubishi-Tanabe Pharma. ST and KT are employees of Mitsubishi-Tanabe Pharma. Nagoya University has filed a patent related to this manuscript: PCT application PCT/JP2022/018823, entitled “PROPHYLACTIC AND/OR THERAPEUTIC AGENT FOR AMYOTROPHIC LATERAL SCLEROSIS” with DI, YI, and MK as coinventor.

## Funding support

The following organizations provided funding support:

- JSPS KAKENHI (JP21K15677 and JP23K14773 to DI; JP20H00527 and JP23H00420 to MK).
- AMED (JP21wm0425013, JP22nk0101575, JP22am0401007, and JP24wm0625301 to MK; JP21wm0425008 to TY; and JP23bm1423006 to S Yokoi).
- Mitsubishi-Tanabe Pharma.

## Supplementary Material

Supplemental data

Unedited blot and gel images

Supplemental tables 5-9

Supporting data values

## Version 1 06/11/2026

In-Press Preview

## Version 2 08/10/2026

Electronic publication
