# Supplementary material for: Fatty acid amide hydrolase inhibition for treatment of amyotrophic lateral sclerosis
Source: JCI Insight. 2026 Jun 11;11(15):e198842. doi: 10.1172/jci.insight.198842 (PMC13463617; doi:10.1172/jci.insight.198842)
Supplement: Supplementary file 1 [file jciinsight-11-198842-s163.pdf]

Supporting Information for

Fatty acid amide hydrolase inhibition for treatment of amyotrophic lateral sclerosis

Daisuke Ito, Madoka Iida, Yohei Iguchi, Atsushi Hashizume, Shinichiro Yamada, Yoshiyuki Kishimoto, Shota Komori, Kazuki Obara, Shuto Nishisaki, Satoshi Yokoi, Teppei Shimamura, Yuto Takemoto, Masahiro Nakatochi, Tomohiro Akashi, Kunihiko Hinohara, Hyeon-Cheol Lee-Okada, Yohei Okada, Junichi Niwa, Gen Sobue, Shinji Tanaka, Ken Takashina, Takehiko Yokomizo, Masahisa Katsuno

Corresponding author: Masahisa Katsuno (katsuno.masahisa.i1@f.mail.nagoya-u.ac.jp) and Daisuke Ito (ito.daisuke.k4@f.mail.nagoya-u.ac.jp)

This file includes:

- Supplemental Methods
- Supplemental Figures S1 to S17
- Supplemental Tables S1 to S9
- Legends for Datasets S1

Other supporting materials for this manuscript include the following:

- Dataset S1

Supplemental Methods

Lipidomics data analysis

Lipidomics data of murine spinal cords were analyzed using Metaboanalyst 6.0. For multivariate analysis, sPLS-DA was performed to discriminate between wild-type mice, untreated $SOD1^{G93A}$ transgenic mice and $SOD1^{G93A}$ transgenic mice treated with PF-04457845. Loading plots indicated the importance of metabolites for sPLS-DA. The concentrations of *N*-acyl taurines and *N*-acyl ethanolamines were log-transformed for One-way ANOVA and Tukey's post-hoc analysis.

RNA-Seq of murine spinal cords

Total RNA was extracted from thoracic spinal cords using the TRIzol™ Plus RNA Purification Kit (Invitrogen). RNA concentration was measured with Quant-iT RiboGreen (Invitrogen), and integrity assessed using the Agilent TapeStation; only samples with RIN >7.0 were used. Libraries were prepared from 1 µg total RNA using the TruSeq Stranded mRNA Sample Prep Kit (Illumina). Poly-A RNA was enriched with poly-T magnetic beads, fragmented, and reverse transcribed into cDNA. After second-strand synthesis, end repair, A-tailing, and adaptor ligation were performed. Libraries were PCR-amplified, quantified with the KAPA qPCR kit, and quality-checked on a TapeStation. Sequencing was performed as paired-end 2×100 bp runs on an Illumina NovaSeq (Macrogen Inc.).

RNA-Seq data analysis

Raw paired-end reads were quality-checked with FastQC (v0.11.5) and trimmed using Trimmomatic (v0.38) with standard parameters. Trimmed reads were aligned to the reference genome using HISAT2 (v2.1.0), and .sam files were converted to .bam files with samtools. Gene-level counts were obtained using featureCounts (v1.6.3) and normalized to TPM. Differentially expressed genes (DEGs) were identified with DESeq2 (v1.24.0) using thresholds of fold change >1.5 and $p < 0.05$. Gene set enrichment was performed with Metascape ($p < 0.01$, min count 3, enrichment factor >1.5), and pathway analysis was conducted using Ingenuity Pathway Analysis (IPA; QIAGEN).

Single-nucleus RNA-Seq of murine spinal cords

Nuclei isolation: Spinal cords from 4 mice for each condition were pooled for homogenization and nuclear isolation. Briefly, fresh frozen murine spinal cord was placed in a Dounce homogenizer (Kimble Chase 2 ml Tissue Grinder) containing ice-cold lysis buffer (10 mM Tris-HCl pH 7.4, 10 mM NaCl, 3 mM MgCl2, 0.1% Igepal and 0.2 U/µl RNase inhibitor). After homogenizing with 5 strokes of pestle A and 5–10 strokes of pestle B, the lysate was diluted with 1.4 mL of lysis buffer and incubated for 5 minutes. The homogenate was passed over a 70 µm strainer (Fisher Scientific). The filtered lysate was centrifuged at 500 × g for 5 min at 4 °C. After centrifugation, the pellet was resuspended in 1.5 ml lysis buffer and incubated for 3 minutes on ice. Then, the pellet was centrifuged again at 500 × g for 5 minutes and resuspended in 1500 µl of wash buffer (1× PBS with 1% BSA and 0.2 U/µl SUPERase-InTM RNase Inhibitor (Thermo Fisher). The nuclei were washed and centrifuged at 500 × g for 5 minutes three times and filtered through a 40 µm cell strainer. The cell suspensions were processed according to the debris removal solution (Miltenyi Biotec) protocol, a density gradient method to remove dead cells and debris. Cells were resuspended in cold PBS with 0.04% BSA and stained with DAPI. The nuclei were sorted with a FACS Aria instrument (Becton Dickson) with a 100 nm nozzle and 405 nm excitation laser. The instrument was controlled by a PC running FACS DiVa™ software (Becton Dickson). These nuclei were centrifuged, inspected for appearance, and concentrated for the next step.

Supplemental Figure S1. One-way ANOVA of metabolomic profiles among Rapid ALS, Slow ALS, and healthy controls.

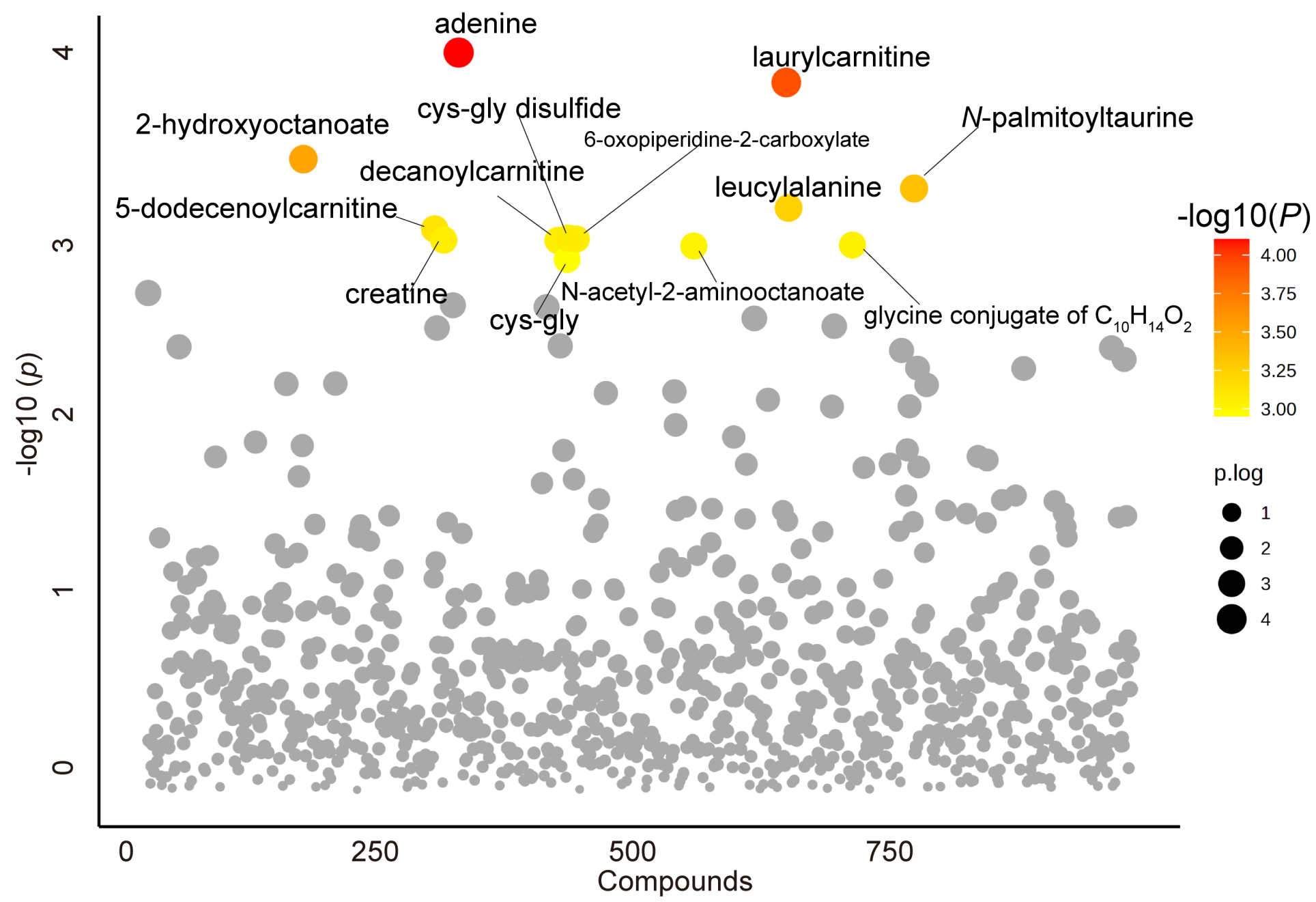

Manhattan plot of metabolomics by ultrahigh-performance liquid chromatography-tandem mass spectrometry (UPLC–MS/MS). Serum metabolites with different levels among Rapid ALS, Slow ALS, and healthy controls determined by MetaboAnalyst 6.0 are indicated in colored circles (FDR < 0.1, one-way ANOVA). cys-gly; cysteinylglycine

Supplemental Figure S2. Heatmap analysis of Rapid ALS and Slow ALS

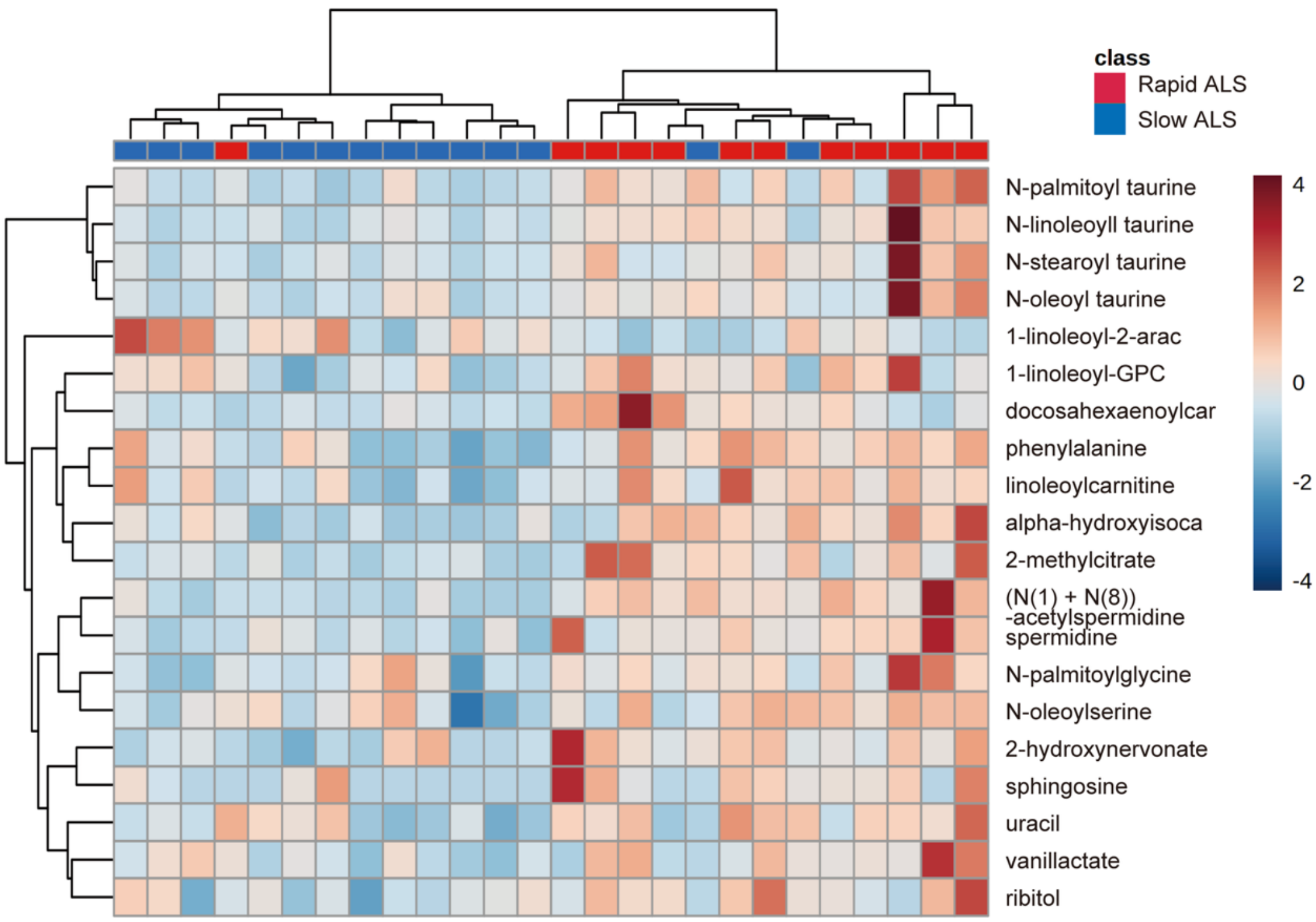

Heatmap analysis comparing Rapid ALS and Slow ALS was performed using top 20 metabolites identified in unpaired *t*-test.

Supplemental Figure S3. Enrichment by subclass of metabolites compared between Rapid ALS vs Slow ALS

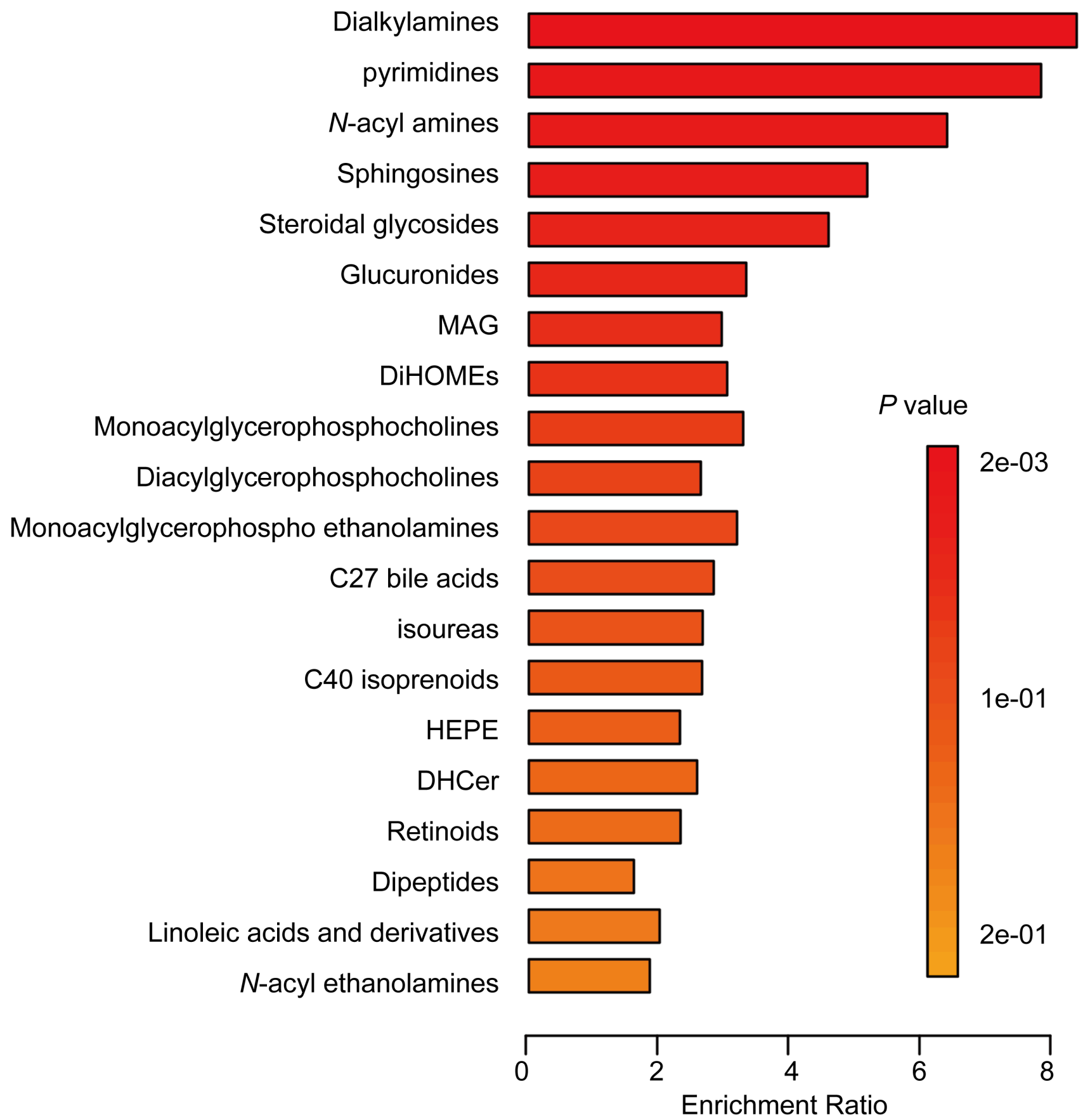

Enrichment analysis between Rapid ALS and Slow ALS by using sub-class of chemical structures to which metabolites belong was performed. Top 20 subclasses of chemical structures are shown.

Supplemental Figure S4. Enrichment by metabolic pathways of metabolites compared between Rapid ALS and Slow ALS

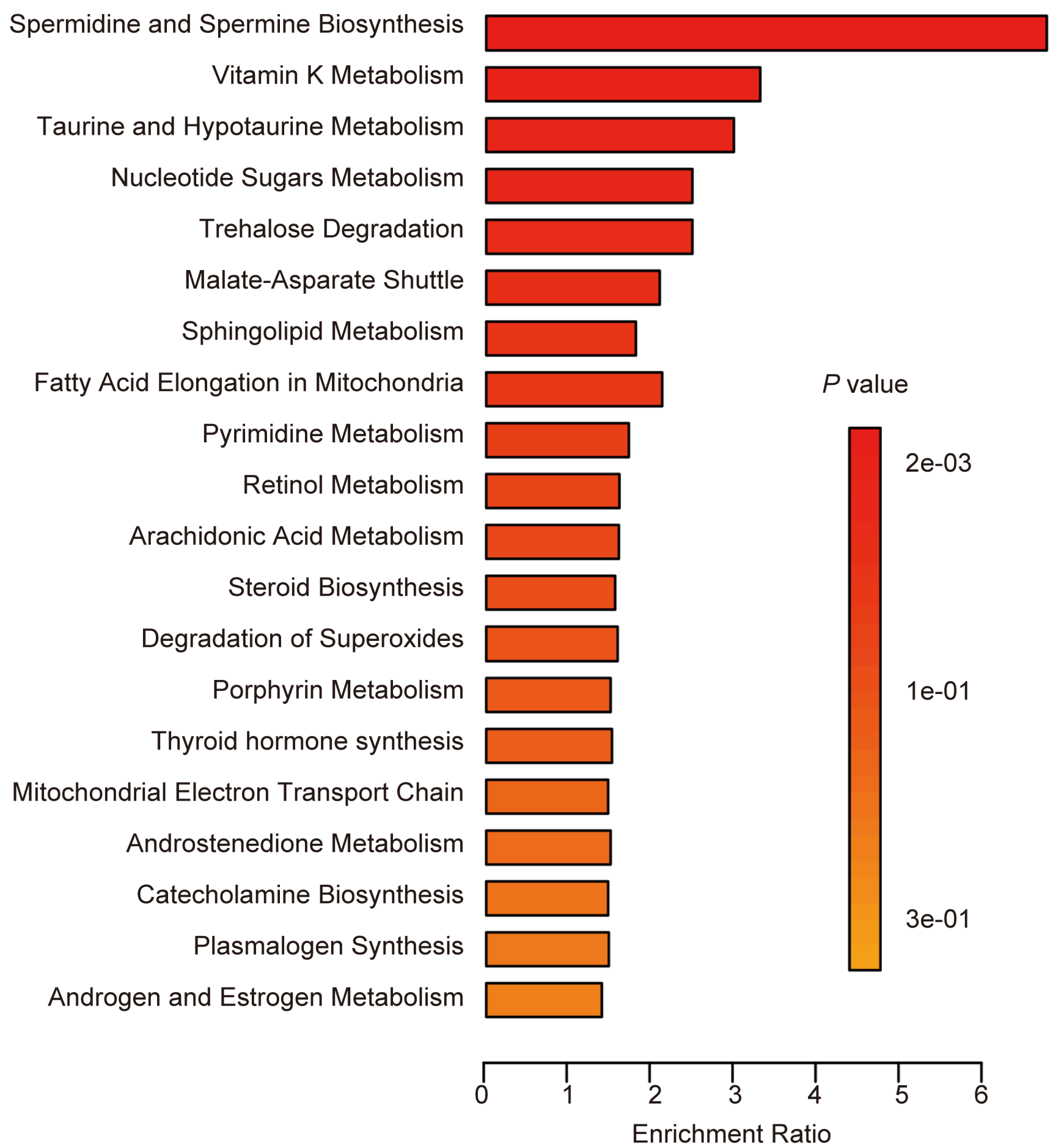

Enrichment analysis between Rapid ALS and Slow ALS by using small molecule pathway database (SMPDB) was performed. Top 20 metabolic pathways are shown.

Supplemental Figure S5. Alteration in N-acyl taurine metabolism among patients with rapidly progressive ALS, patients with slowly progressive ALS, and healthy individuals, and their correlation with disease progression in the discovery and replication cohorts, including sex-stratified analysis (male)

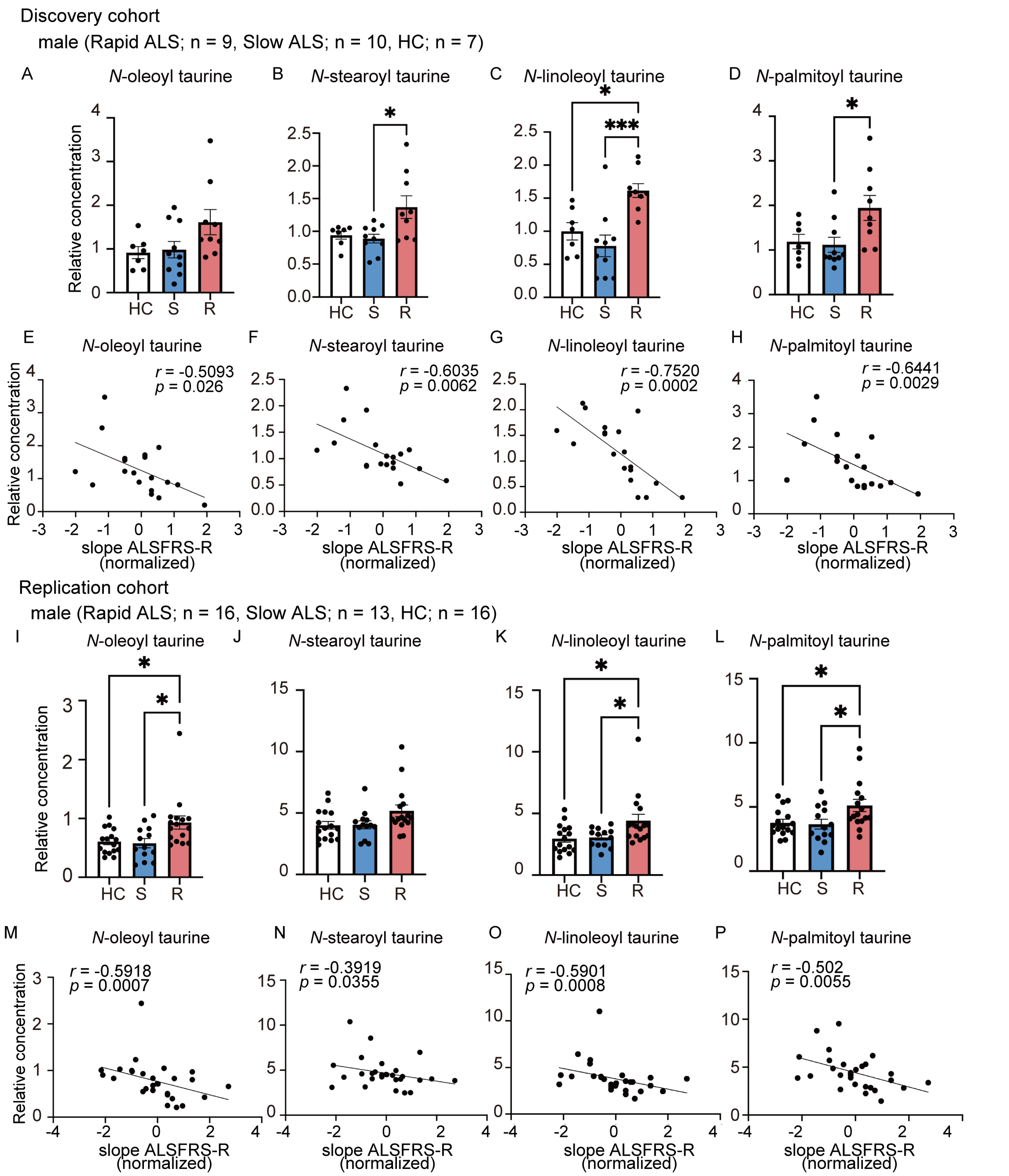

Male subjects in the discovery cohort (Rapid ALS, n = 9; Slow ALS, n = 10; healthy controls [HC], n = 7) and the replication cohort (Rapid ALS, n = 16; Slow ALS, n = 13; HC, n = 16) were analyzed. (**A–D**) Serum levels of *N*-acyl taurine metabolism of male subjects in the discovery cohort (A, *N*-oleoyl taurine; B, *N*-stearoyl taurine; C, *N*-linoleoyl taurine; D, *N*-palmitoyl

taurine). (**E–H**) Correlation between the serum levels of *N*-acyl taurines and the prospective, longitudinal change in ALSFRS-R between the first and second evaluations (slope ALSFRS-R) in the discovery cohort (E, *N*-oleoyl taurine; F, *N*-stearoyl taurine; G, *N*-linoleoyl taurine; H, *N*-palmitoyl taurine). (**I–L**) Serum levels of *N*-acyl taurine metabolism in the replication cohort (I, *N*-oleoyl taurine; J, *N*-stearoyl taurine; K, *N*-linoleoyl taurine; L, *N*-palmitoyl taurine). (**M–P**) Correlation between the serum levels of N-acyl taurines and slope ALSFRS-R in the replication cohort (M, *N*-oleoyl taurine; N, *N*-stearoyl taurine; O, *N*-linoleoyl taurine; P, *N*-palmitoyl taurine). ALSFRS-R slope values were transformed using the Yeo–Johnson transformation before correlation analysis. One-way ANOVA and Tukey's post hoc analysis were performed (*$p < 0.05$, **$p < 0.01$ and ***$p < 0.001$). HC, healthy controls; R, Rapid ALS; S, Slow ALS. A coefficient value (r) of 0.40–0.59 is considered moderate, while 0.60–0.79 is considered strong in Spearman's rank correlation coefficient. Error bars indicate the SEM.

Supplemental Figure S6. Alteration in N-acyl taurine metabolism among patients with rapidly progressive ALS, patients with slowly progressive ALS, and healthy individuals, and their correlation with disease progression in the discovery and replication cohorts, including sex-stratified analysis (female)

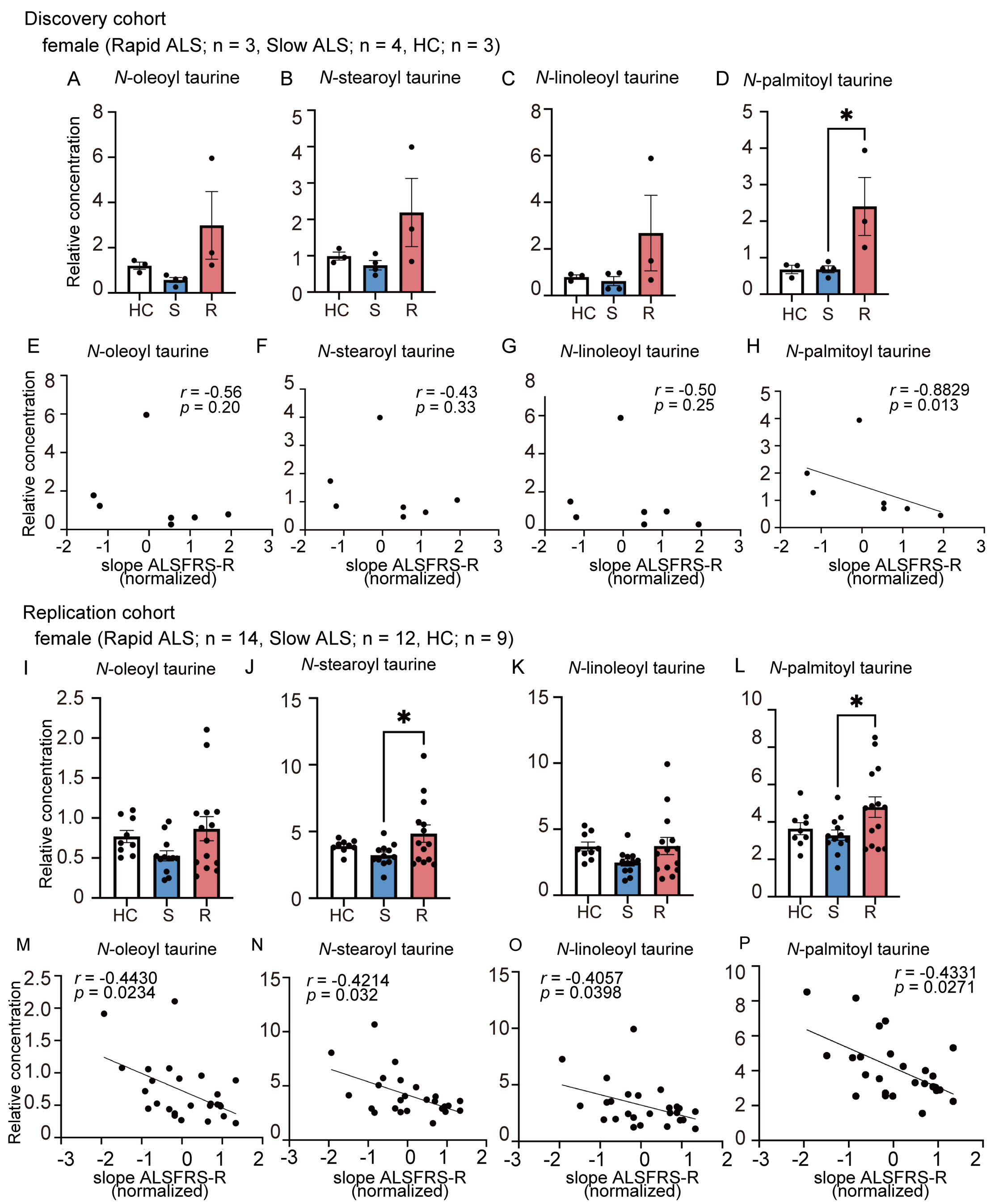

Female subjects in the discovery cohort (Rapid ALS, n = 3; Slow ALS, n = 4; healthy controls [HC], n = 3) and the replication cohort (Rapid ALS, n = 14; Slow ALS, n = 12; HC, n =

9) were analyzed. (**A–D**) Serum levels of *N*-acyl taurine metabolism of female subjects in the discovery cohort (A, *N*-oleoyl taurine; B, *N*-stearoyl taurine; C, *N*-linoleoyl taurine; D, *N*-palmitoyl taurine). (**E–H**) Correlation between the serum levels of *N*-acyl taurines and the prospective, longitudinal change in ALSFRS-R between the first and second evaluations (slope ALSFRS-R) in the discovery cohort (E, *N*-oleoyl taurine; F, *N*-stearoyl taurine; G, *N*-linoleoyl taurine; H, *N*-palmitoyl taurine). (**I–L**) Serum levels of *N*-acyl taurine metabolism in the replication cohort (I, *N*-oleoyl taurine; J, *N*-stearoyl taurine; K, *N*-linoleoyl taurine; L, *N*-palmitoyl taurine). (**M–P**) Correlation between the serum levels of *N*-acyl taurines and slope ALSFRS-R in the replication cohort (M, *N*-oleoyl taurine; N, *N*-stearoyl taurine; O, *N*-linoleoyl taurine; P, *N*-palmitoyl taurine). ALSFRS-R slope values were transformed using the Yeo–Johnson transformation before correlation analysis. One-way ANOVA and Tukey's post hoc analysis were performed (*$p < 0.05$). HC, healthy controls; R, Rapid ALS; S, Slow ALS. A coefficient value (r) of 0.40–0.59 is considered moderate, while 0.60–0.79 is considered strong in Spearman's rank correlation coefficient. Error bars indicate the SEM.

Supplemental Figure S7. Serum levels of *N*-acyl ethanolamines detected in subjects with amyotrophic lateral sclerosis and healthy individuals

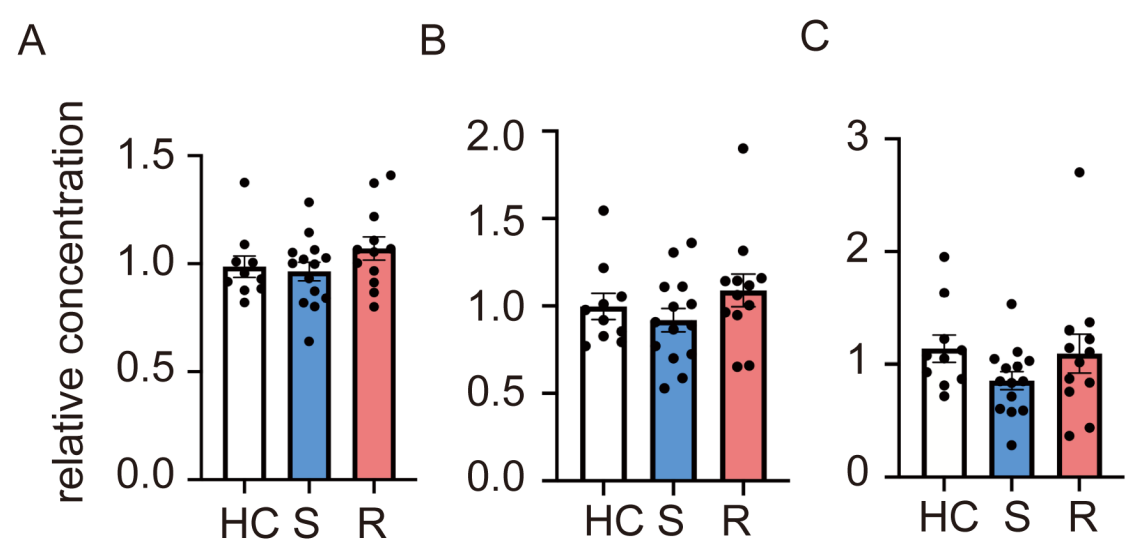

Serum levels of (**A**) palmitoyl ethanolamide, (**B**) oleoyl ethanolamide, and (**C**) linoleoyl ethanolamide. One-way ANOVA and Tukey's post hoc analysis were performed. There was no significant difference among the three groups. HC, healthy controls; R, Rapid ALS; S, Slow ALS. Error bars indicate the SEM.

Supplemental Figure S8. PF-04457845 does not affect cell viability or LDH release in mock-transfected NSC-34 cells.

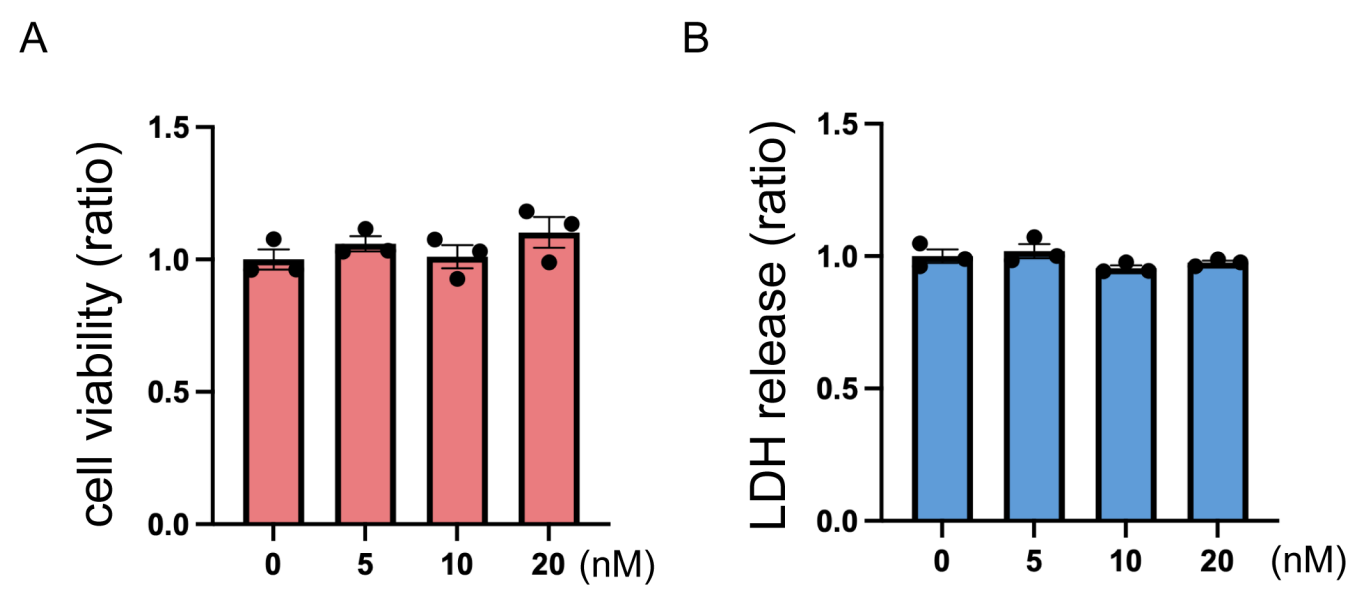

PF-04457845 was administered in mock-transfected NSC-34 cells as a control. PF-04457845 did not show significant effects in (**A**) WST assay or (**B**) LDH release. One-way ANOVA followed by Dunnett's multiple-comparisons test was performed, with each concentration compared with the baseline condition. Error bars indicate the SEM.

Supplemental Figure S9. PF-04457845 does not exert protective effects in control iPSC-derived motor neurons

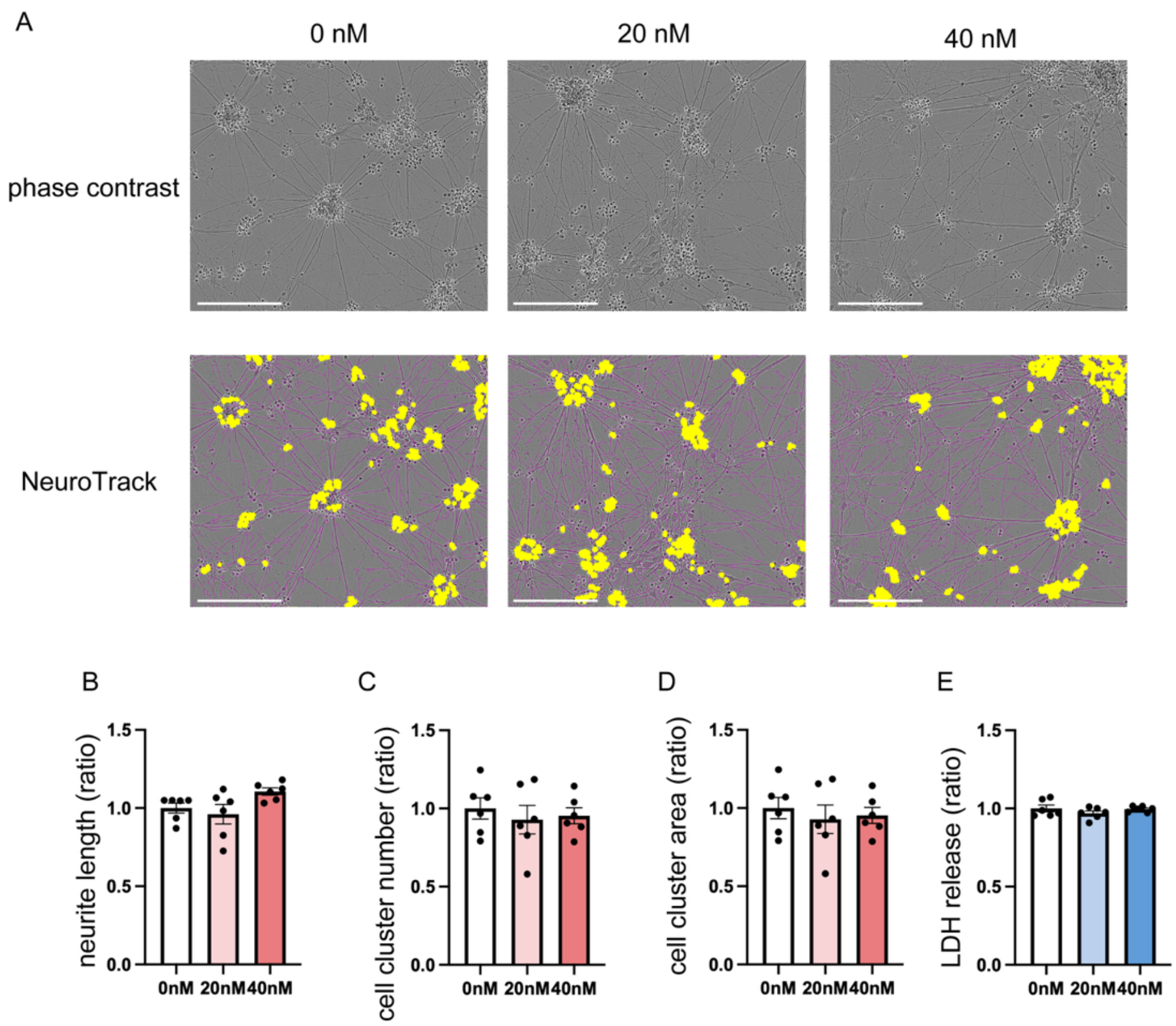

(**A**) Analysis of cell morphology by IncuCyte SX5®. iPS-MNs from a healthy control were captured by phase separation and neurites (purple) and cell clusters (yellow) were automatically recognized by NeuroTrack algorithm. (**B**) Neurite length (n = 6 each from iPS-MN derived from one healthy individual). (**C**) Number of cell clusters (n = 6 from iPS-MN derived from one healthy individual). (**D**) Area of cell clusters (n = 6 from iPS-MN derived from one healthy individual). (**E**) LDH assay (n = 6 from iPS-MN derived from one healthy individual). PF-04457845 did not significantly alter neuronal morphology or LDH release in control iPSC-derived motor neurons. One-way ANOVA was performed. Scale bars: 200 μm. Error bars indicate the SEM.

Supplemental Figure S10. In vivo analysis of PF-04457845 in $SOD1^{G93A}$ transgenic mice with sex-specific stratification.

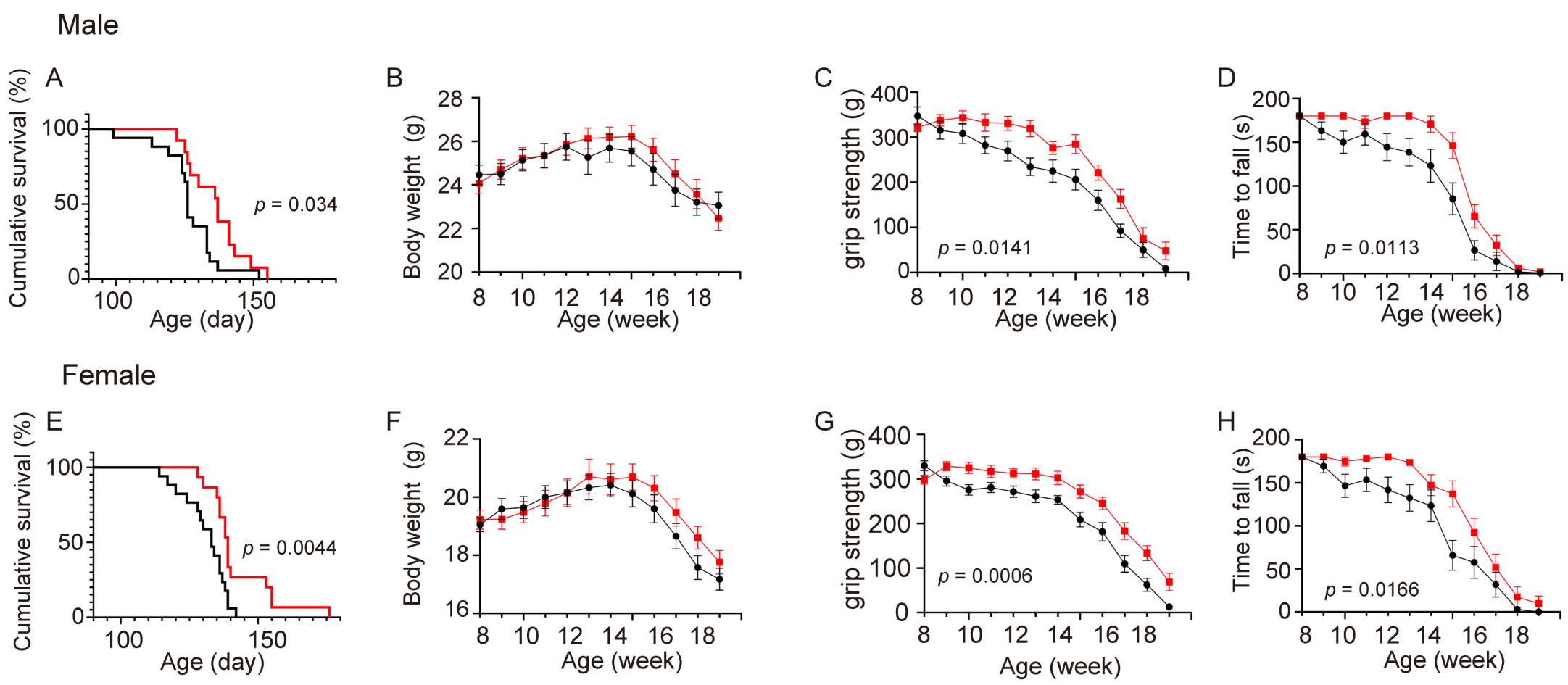

(**A–D**) Male mice: (A) survival (PF-04457845, n = 13, Untreated, n = 17; median survival, 137, 126 days, respectively; $p = 0.034$, log-rank test), (B) body weight ($p = 0.14$, two-way ANOVA), (C) grip strength ($p = 0.0141$, two-way ANOVA) and (D) rotarod test ($p = 0.0113$, two-way ANOVA). (**E–H**) Female mice: (E) survival (PF-04457845, n = 15, Untreated, n = 17; median survival, 139, 133 days, respectively; $p = 0.0044$, log-rank test), (F) body weight ($p = 0.2177$, two-way ANOVA), (G) grip strength ($p = 0.0006$, two-way ANOVA) and (H) rotarod test ($p = 0.0166$, two-way ANOVA). To impute values after the endpoint, the final body weights were carried forward and the values of grip strength and rotarod were imputed to zero. Error bars indicate the SEM.

Supplemental Figure S11. PF-04457845 does not alter body weight or motor performance in C57BL/6 wild-type mice.

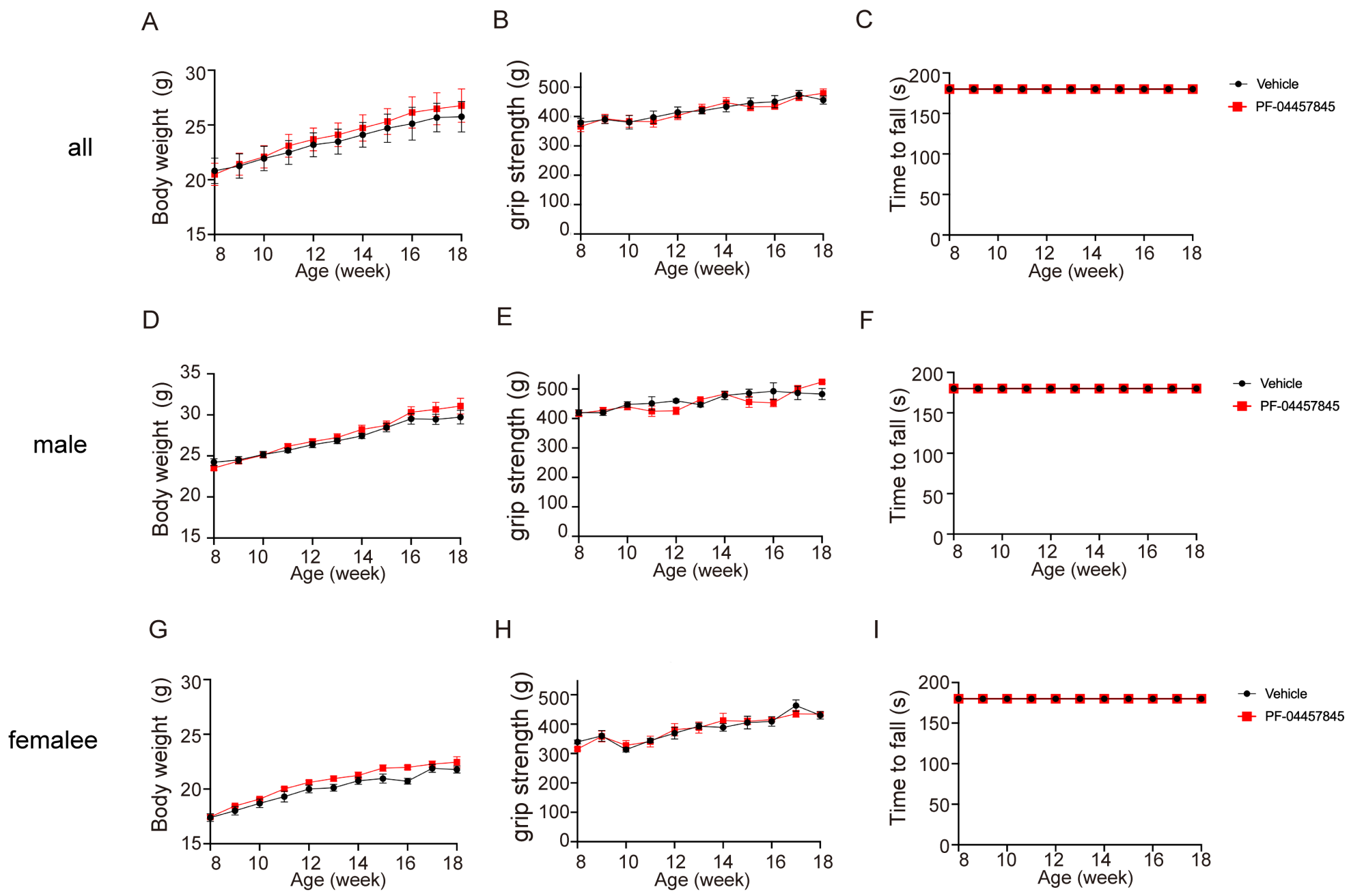

(A) Body weight, **(B)** grip strength, and (**C)** rotarod test in C57BL/6 wild-type mice treated with or without PF-04457845 (PF-04457845, n = 10; male, n = 5; female, n = 5) (vehicle, n = 10; male, n = 5; female, n = 5). Male and female data are shown separately in the lower panels. (**D–F**) Male mice: (D) body weight, (E) grip strength, and (F) rotarod test. (**G–I**) Female mice: (G) body weight, (H) grip strength, and (I) rotarod test, respectively. Data were analyzed by two-way ANOVA, and no significant effect of PF-04457845 treatment was observed. Error bars indicate the SEM.

Supplemental Figure S12. PF-04457845 does not alter body weight or motor performance in B6/SJL wild-type mice

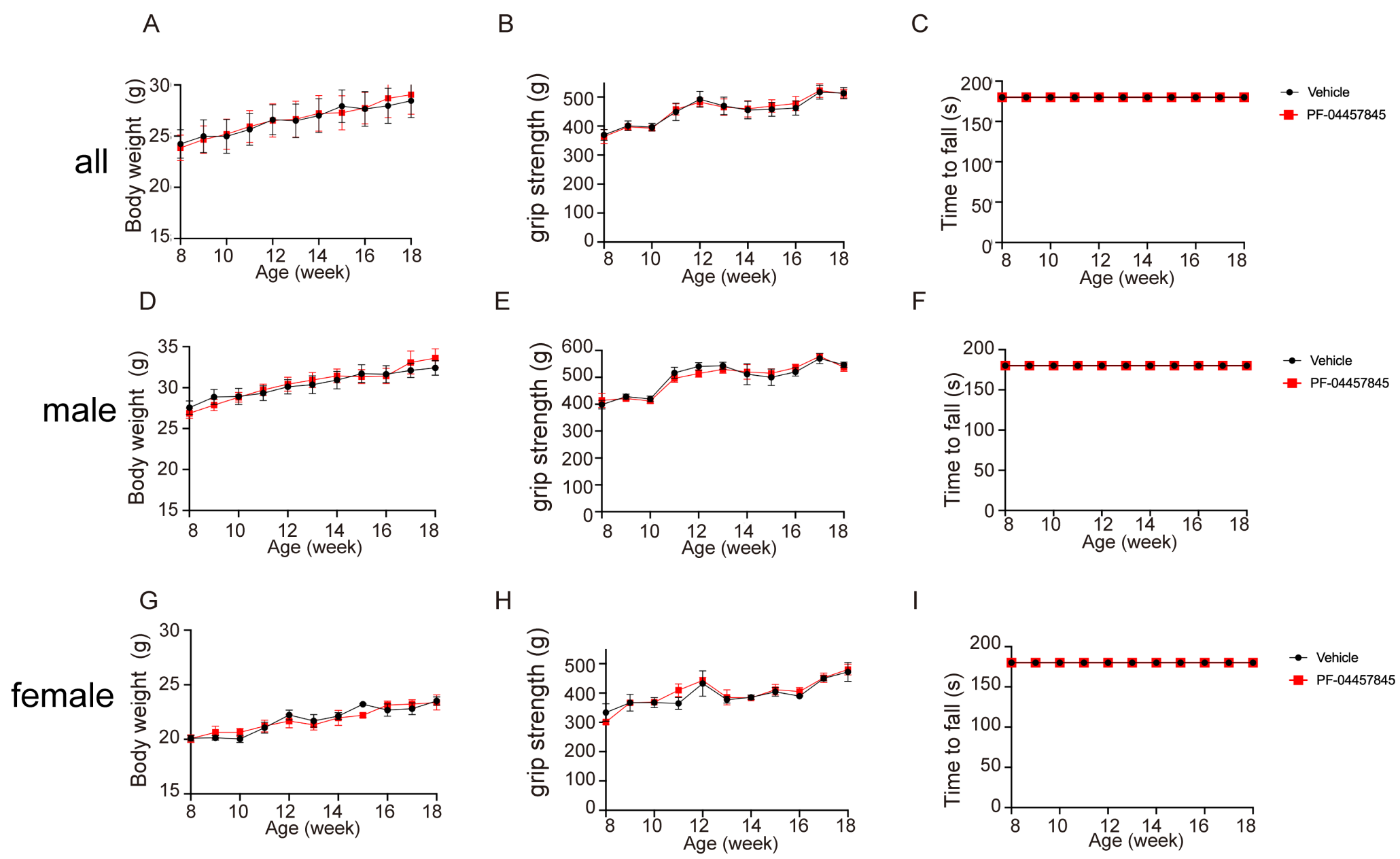

(**A**) Body weight, **(B)** grip strength, and (**C)** rotarod test in B6/SJL wild-type mice treated with or without PF-04457845. (PF-04457845, n = 9; male, n = 5; female, n = 4; vehicle, n = 9, male, n = 5; female, n = 4). Male and female data are shown separately in the lower panels. (**D–F**) Male mice: (D) body weight, (E) grip strength, and (F) rotarod test, (**G–I**) Female mice: (G) body weight, (H) grip strength, and (**I**) rotarod test, respectively. Data were analyzed by two-way ANOVA, and no significant effect of PF-04457845 treatment was observed. Error bars indicate the SEM.

Supplemental Figure S13. Elevated levels of Rapid ALS-related NATs in the spinal cord of SOD1^{G93A} transgenic mice

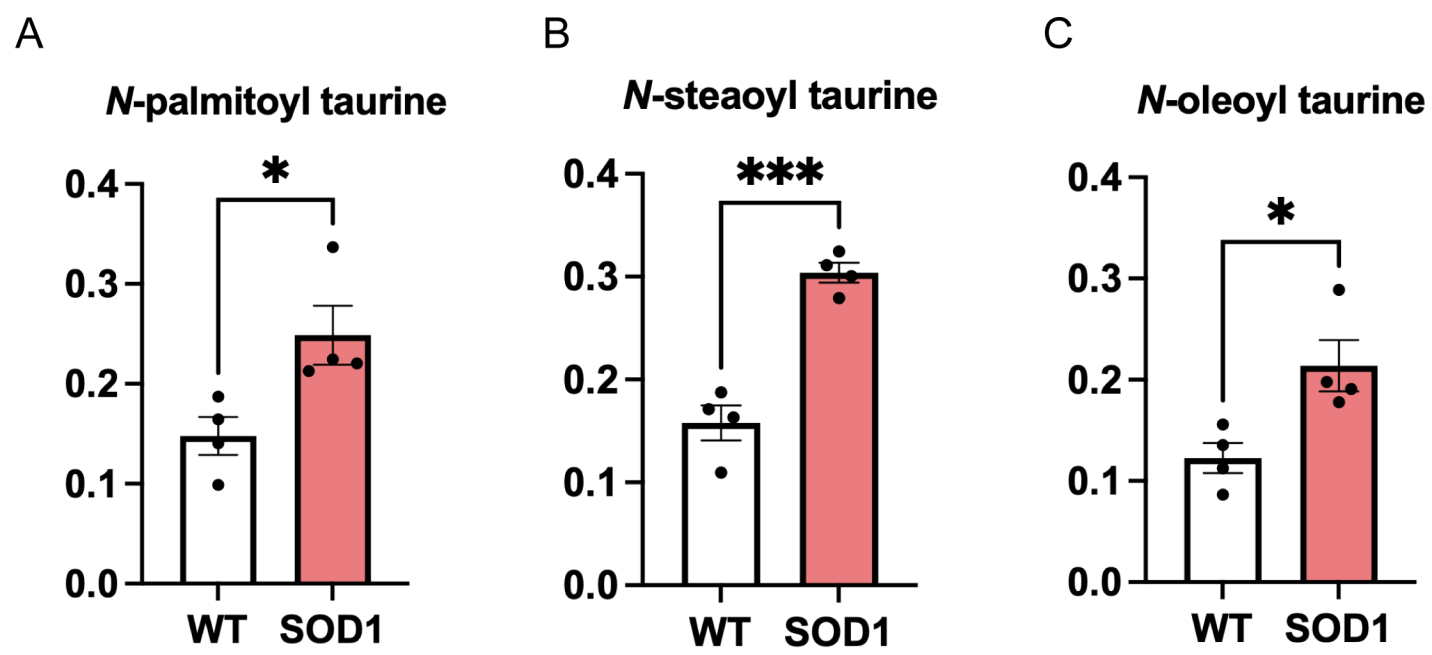

Lipidomic analysis of spinal cord tissue from SOD1^{G93A} transgenic mice (n = 4) and wild-type mice (n = 4) revealed significantly elevated levels of (**A**) *N*-palmitoyl taurine, (**B**) *N*-stearoyl taurine, and (**C**) *N*-oleoyl taurine in the transgenic mice. These three *N*-acyl taurines were among the four species identified in the serum of ALS patients, and their upregulation in SOD1^{G93A} mice is consistent with the elevations observed in Rapid ALS patients (Figure 2). The remaining metabolite, *N*-linoleoyl taurine, was not detected in the lipidomic analysis of the murine spinal cord specimens. Data are presented as mean ± SEM *$p < 0.05$, ***$p < 0.001$ by Student's *t*-test. WT; wild type, SOD1; SOD1^{G93A} transgenic mice

Supplemental Figure S14. Predicted changes in the IL-13 pathway in IPA analysis of spinal cords of SOD1^{G93A} transgenic mice treated with PF-04457845

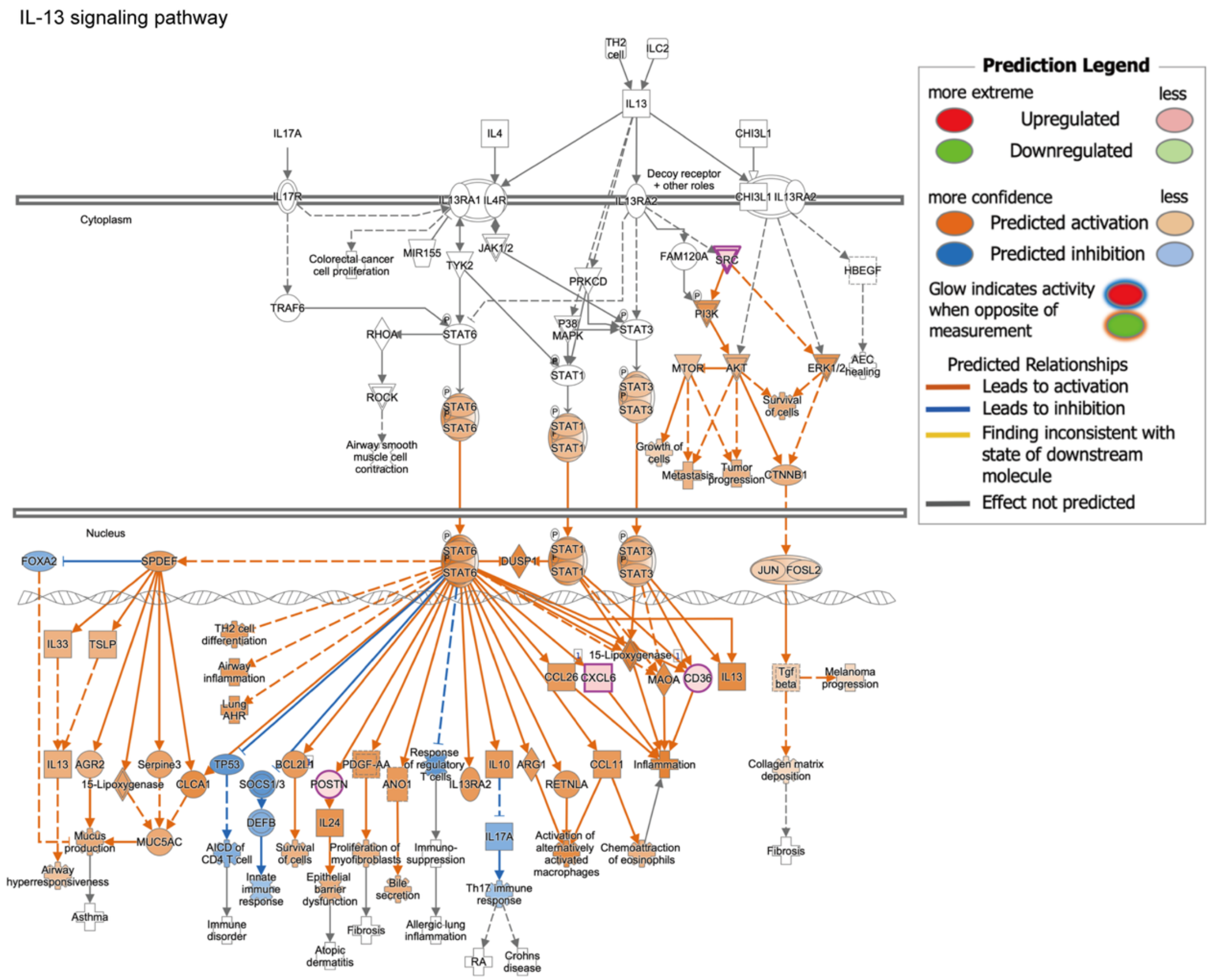

Purple-circled or squared genes were upregulated in the bulk spinal cord RNA-Seq data from SOD1^{G93A} treated with PF-04457845. IPA analysis predicted upregulation and downregulation of genes in IL-13 signaling pathway. Those genes were shown in the IL-13 signaling pathway.

Supplemental Figure S15. Abundance of cannabinoid receptor 1 (CB1)-positive neurons and pathway analysis.

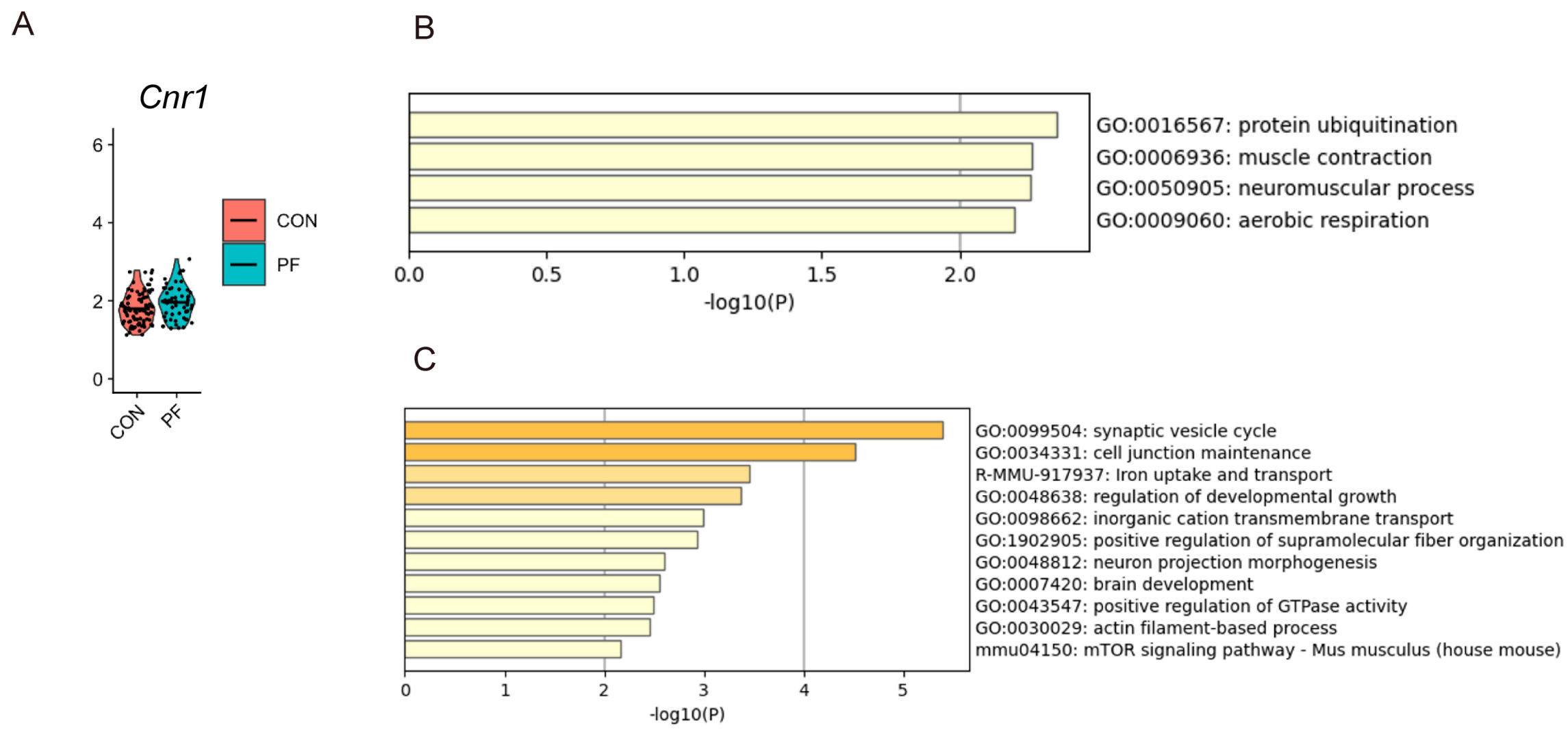

(A) Abundance of cannabinoid receptor 1 (CB1)-positive neurons in PF-04457845-treated and untreated mice. **(B, C)** Pathway analysis of upregulated (B) and downregulated (C) genes in CB1-positive neurons from PF-04457845-treated mice compared with untreated mice.

Supplemental Figure S16. Differentially expressed genes and altered pathways in oligodendrocytes.

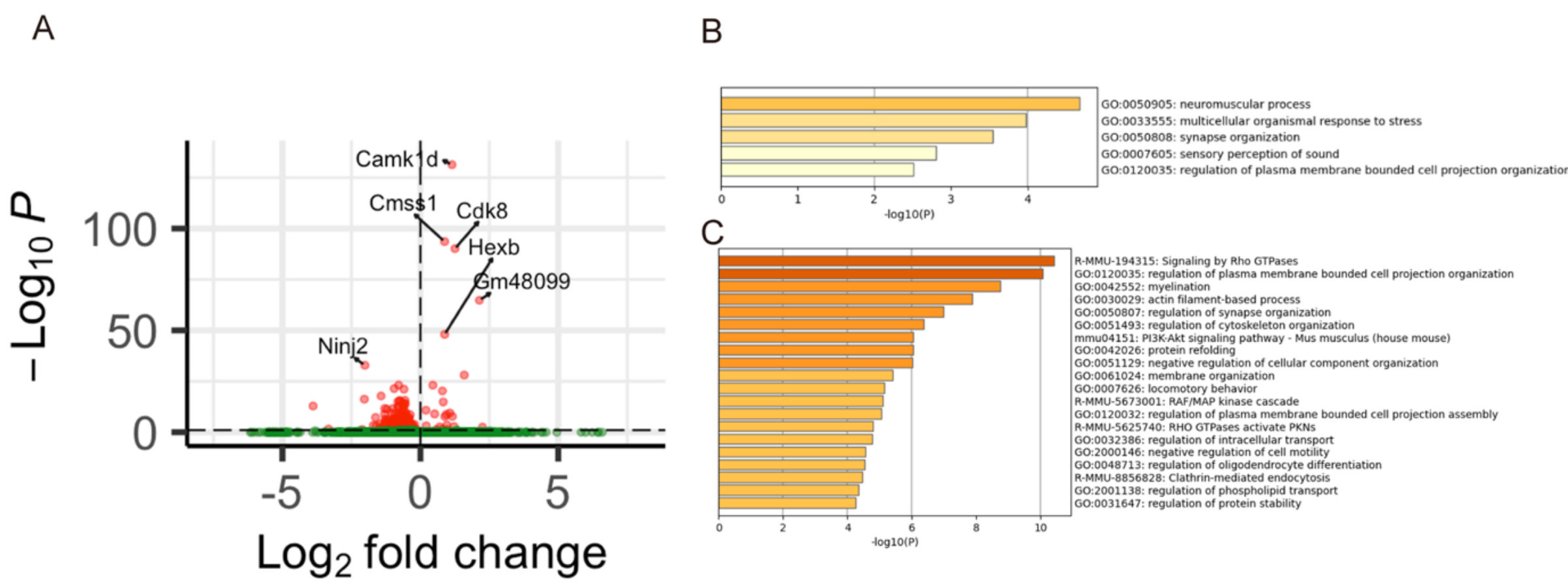

(**A**) Volcano plot of the differentially expressed genes in the oligodendrocyte cluster from mice treated with PF-04457845 compared with untreated mice. (**B**, **C**) Gene Ontology enrichment analysis of upregulated (B) and downregulated (C) genes in the oligodendrocyte cluster from mice treated with PF-04457845 compared with untreated mice.

Supplemental Figure S17. Differentially expressed genes and altered pathways in microglia.

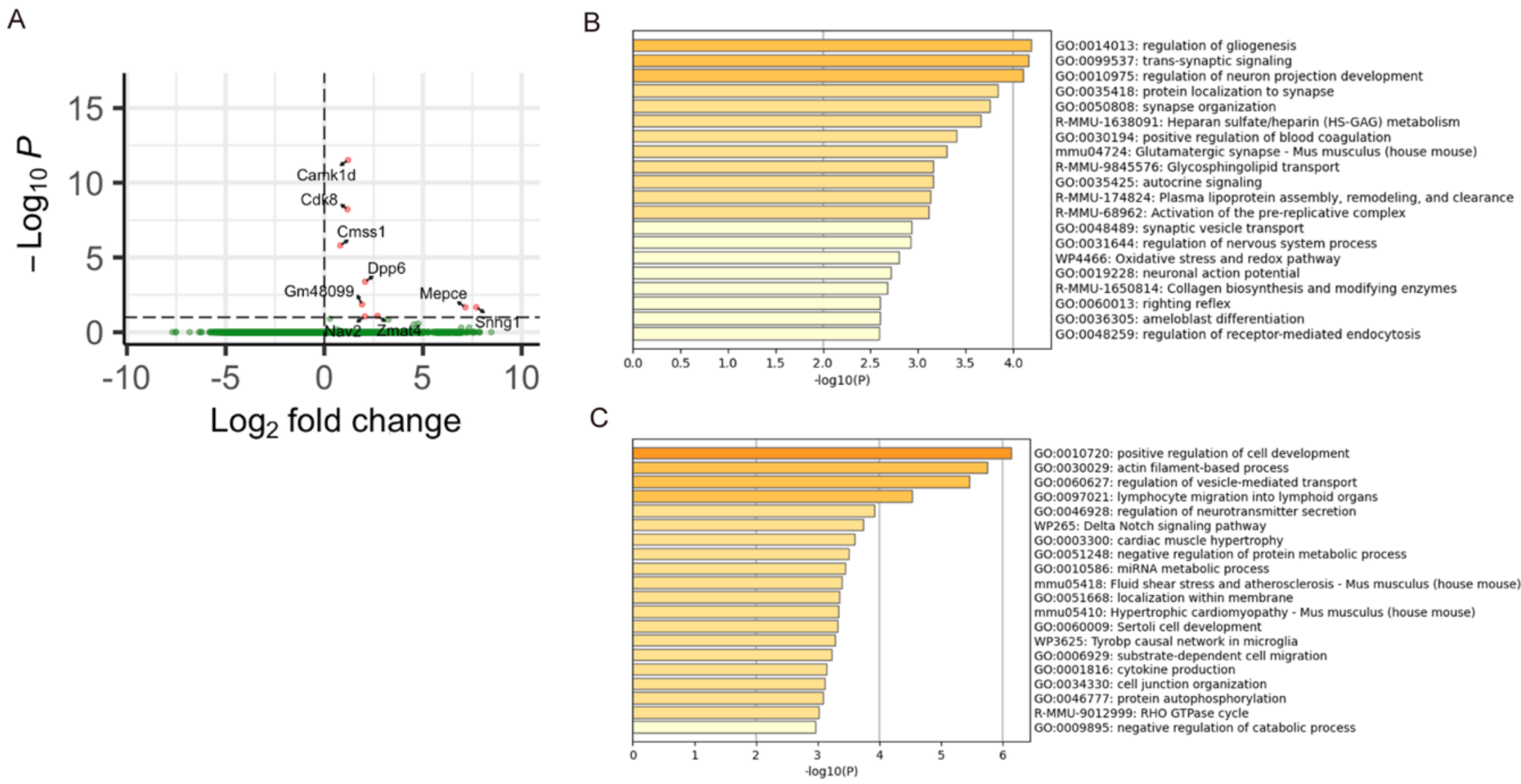

(**A**) Volcano plot of the differentially expressed genes in the microglia cluster from mice treated with PF-04457845 compared with untreated mice. (**B**, **C**) Gene Ontology enrichment analysis of upregulated (B) and downregulated (C) genes in the microglia cluster from mice treated with PF-04457845 compared with untreated mice.

Supplemental Table S1. Baseline characteristics of subjects with ALS and healthy controls in the Discovery cohort

	Rapid ALS n = 12	Slow ALS n = 14	HC n = 10	*p*
Age	68.1 ± 7.3	65.4 ± 9.1	67.5 ± 5.1	N.S.[A]
Sex (Male %)	75	71	70	N.S.[B]
BMI	22.5 ± 2.7	21.4 ± 2.9	22.9 ± 1.8	N.S.[A]
Disease duration (months)	8.8 ± 3.0	12.4 ± 5.4		N.S. [C]
Onset site (bulbar %)	33.3	42.9		N.S. [B]
ALSFRS-R at the initial evaluation	39.2 ± 4.6	41.0 ± 5.4		N.S. [C]
ΔALSFRS-R (Pre) (/month)	-1.05	-0.63		0.026 [C]
ΔALSFRS-R (Post) (/month)	-2.07	-0.25		< 0.001 [C]
%FVC	91.1 ± 15.8	102.2 ± 22.1		N.S.[C]
MMSE	27.6 ± 1.4	27.7 ± 1.3		N.S.[C]
Riluzole	11 (91.7%)	12 (85.7%)		N.S.[B]
Edaravone	2 (16.7%)	5 (35.7%)		N.S.[B]

Data are shown as mean ± standard deviation.

ALSFRS-R, ALS functional rating scale-revised; ΔALSFRS-R (Pre), the slope of ALSFRS-R between the disease onset and the first evaluation; ΔALSFRS-R (Post), the difference of ALSFRS-R between the first evaluation and the second evaluation; HC, healthy controls; MMSE, Mini-Mental State examination; N.S., not significant.

[A] one-way ANOVA for 3 groups comparison; [B] *Chi*-squared test ; [C] unpaired *t*-test for 2 groups comparison

Supplemental Table S2. Baseline characteristics of subjects with ALS and healthy controls in the replication cohort

	Rapid ALS n = 30	Slow ALS n = 25	HC n = 25	*p*
Age	64.1 ± 10.8	64.2 ± 8.7	62.8 ± 7.8	N.S.[A]
Sex (Male %)	53	52	64	N.S.[B]
BMI	21.0 ± 3.5	21.1 ± 3.0	23.6 ± 2.6	0.003[C]
Disease duration (months)	10.5 ± 4.3	11.9 ± 5.6		N.S. [D]
Onset site (bulbar %)	16.7	28.0		N.S. [B]
ALSFRS-R at the initial evaluation	40.8 ± 3.9	42.2 ± 3.2		N.S. [D]
slope ALSFRS-R (Pre) (/month)	-0.79 ± 0.56	-0.59 ± 0.36		N.S. [D]
slope ALSFRS-R (Post) (/month)	-2.39 ± 2.25	-0.23 ± 0.24		< 0.001 [D]
%FVC	88.8 ± 26.7	98.4 ± 19.9		N.S.[C]
MMSE	28.5 ± 1.6 (n = 26)	27.9 ± 1.9 (n = 24)		N.S.[C]
Riluzole	26 (86.7 %)	22 (88.0%)		N.S.[B]
Edaravone	20 (66.7 %)	21 (84.0%)		N.S.[B]

Data are shown mean ± standard deviation.

ALSFRS-R, ALS functional rating scale-revised; slope ALSFRS-R (Pre), the slope of ALSFRS-R between the disease onset and the first evaluation; slope ALSFRS-R (Post), the difference of ALSFRS-R between the first evaluation and the second evaluation; HC, healthy controls; MMSE, mini mental scale examination; N.S., not significant.

[A] one-way ANOVA for 3 groups comparison; [B] *Chi*-squared test ; [C] one-way ANOVA followed by Tukey's post-hoc analysis: Rapid ALS vs. Slow ALS, not significant; Rapid ALS vs. HC, p = 0.006; Slow ALS vs. HC, p = 0.014; [D] unpaired *t*-test for 2 groups comparison

Supplemental Table S3. Significantly different serum metabolites among Rapid ALS, Slow ALS and healthy controls by one-way ANOVA

	-log10*(p)*	FDR
Adenine	4.1041	0.049763
Laurylcarnitine (C12)	3.9376	0.049763
2-hydroxyoctanoate	3.5107	0.067474
N-palmitoyl taurine	3.3472	0.067474
Leucylalanine	3.2392	0.067474
5-dodecenoylcarnitine (C12:1)	3.1224	0.067474
Cysteinylglycine disulfide	3.0711	0.067474
Decanoylcarnitine (C10)	3.0661	0.067474
6-oxopiperidine-2-carboxylate	3.0606	0.067474
Creatine	3.0582	0.067474
N-acetyl-2-aminooctanoate	3.0325	0.067474
Glycine conjugate of $C_{10}H_{14}O_2$	3.0272	0.067474
Cysteinylglycine	2.9524	0.073992

FDR, false discovery rate.

One-way ANOVA was performed on serum metabolomics to compare Rapid ALS, Slow ALS, and healthy controls using Metaboanalyst 6.0. Thirteen metabolites showed an FDR below 0.1.

Supplemental Table S4. Results of metabolome analysis comparing Rapid ALS and Slow ALS

metabolite	pathway	log2(FC)	-log10(*p*)
N-palmitoyl taurine	Endocannabinoid	1.0535	2.922
Spermidine	Polyamine	1.0881	2.701
N-linoleoyl taurine	Endocannabinoid	1.3591	2.2603
N-stearoyl taurine	Endocannabinoid	0.89623	2.1383
docosahexaenoylcarnitine	Acylcarnitine	1.0101	1.8431
N-oleoyl taurine	Endocannabinoid	1.1747	1.7922
Sphingosine	Sphingolipid Metabolism	0.8017	1.7049
Daidzein sulfate	Food Component/Plant	1.7907	1.5593
Indoleacetylcarnitine	Acylcarnitine	0.92002	1.5498
Pregnanediol-3-glucuronide	Progestin Steroids	0.7215	1.533
Adipoylcarnitine	Acylcarnitine	1.0382	1.5042
9,10-DiHOME	Fatty Acid, Dihydroxy	0.60084	1.4469
Octadecenedioylcarnitine	Acylcarnitine	0.916	1.4433
Heptenedioate	Fatty Acid, Dicarboxylate	0.90251	1.4136
2-oleoylglycerol	Fatty Acid, Dicarboxylate	0.8933	1.4135
maltose	Glycogen Metabolism	0.69623	1.3879
Histidylalanine	Dipeptide	0.69425	1.3657
Glycochenodeoxycholate glucuronide	Primary Bile Acid Metabolism	1.3739	1.3503
5-HEPE	Eicosanoid	1.1095	1.3478
Suberoylcarnitine	Acylcarnitine	1.2586	1.3385

FC, fold change. Differences in serum metabolites between Rapid ALS and Slow ALS were analyzed by unpaired *t*-test using Metaboanalyst 6.0. Metabolites with fold change (FC) > 1.5 and *p*-value < 0.05 in *t*-test are shown.

Supplemental Table S5. Metabolites in focused metabolic pathways

Table S5 is shown in Dataset S1 as an Excel table.

Supplemental Table S6. Cell viability assay of ALS cell models treated with chemical compounds

Compound	Metabolic Pathway	Viability[A] ($mTDP43^{A315T}$)	Concentration[B] (μM)	Viability[A] ($mSOD1^{G93A}$)	Concentration[B] (μM)
Clofibrate	β oxidation	1.06	10	1.05	1
Metformin	β oxidation	1	0	1.07	0.1
Oleic acid	β oxidation	1.1	10	1	0
Riboflavin	β oxidation	1.21	10	1.11	10
Trimetazidine	β oxidation	1	0	1.12	1
Allantoin	Purine/Xanthine	1.58	10	1.31	0.1
Febuxostat	Purine/Xanthine	1.06	0.1	1.07	0.1
Inosine	Purine/Xanthine	1.01	1	1.01	0.1
Theobromine	Purine/Xanthine	1.27	10	1.12	0.1
Xanthine	Purine/Xanthine	1.08	10	1.06	1
Xanthosine	Purine/Xanthine	1.24	0.1	1	0
Agmatine	Polyamine	1.26	10	1.24	10
Arginine	Polyamine	1.28	1	1	0
Eflornithine	Polyamine	1.56	1	1.02	10
MDL72527	Polyamine	1	0	1.01	10
Mitquazone	Polyamine	1.07	10	1.02	1
Ornithine	Polyamine	1.13	10	1.22	10
S-adenosyl-L-methionine	Polyamine	1	0	1	0
Spermidine	Polyamine	1.12	1	1.08	0.1
Spermine	Polyamine	1	0	1.04	1
2-arachidonoylglycerol	Endocannabinoid	1.29	10	1.04	10
Arachidonoylethanolamide	Endocannabinoid	1.4	10	1.12	1
Arvanil	Endocannabinoid	1.05	0.1	1.12	10
PF-04457845	Endocannabinoid	1.26	20 nM	1.26	10 nM
N-palmitoyl taurine	*N*-acyl taurine	1.29	10	1.21	10
N-stearoyl taurine	*N*-acyl taurine	1.28	10	1.23	10
N-oleoyl taurine	*N*-acyl taurine	1.14	10	1.32	10
Glycocholic acid	Bile acid	1.03	0.1	1.04	0.1
Glycoursodeoxycholic acid	Bile acid	1.13	10	1.0	0
Riluzole	Control	1.03	0.1	1.0	0
Edaravone	Control	1.2	1	1.1	10

[A]The ratio of cell viability to that with vehicle treatment at the most effective concentration.
[B]The most effective concentration at which the cell viability was the highest in this assay.
Compounds were administered at concentrations of 0, 0.1, 1, or 10 μM, except for PF-04457845 which was administered at 0, 5, 10, and 20 nM considering its IC_{50}.

Supplemental Table S7. Raw data of Lipidomics analysis of murine spinal cords.

Table S7 is shown in Dataset S1 as an Excel table.

Supplemental Table S8. Differential gene expression analysis of RNA sequence in spinal cords of SOD1^{G93A} transgenic mice treated with PF-04457845

Table S8 is shown in Dataset S1 as an Excel table.

Supplemental Table S9. Differential gene expression analysis of single-nucleus RNA-Seq on a neuron cluster of spinal cords of SOD1^{G93A} transgenic mice treated with PF-04457845

Table S9 is shown in Dataset S1 as an Excel table.
