# Supplementary material for: Fatty acid amide hydrolase inhibition for treatment of amyotrophic lateral sclerosis
Source: JCI Insight. 2026 Jun 11;11(15):e198842. doi: 10.1172/jci.insight.198842 (PMC13463617; doi:10.1172/jci.insight.198842)
Supplement: Supplementary file 2 [file jciinsight-11-198842-s164.pdf]

CD36

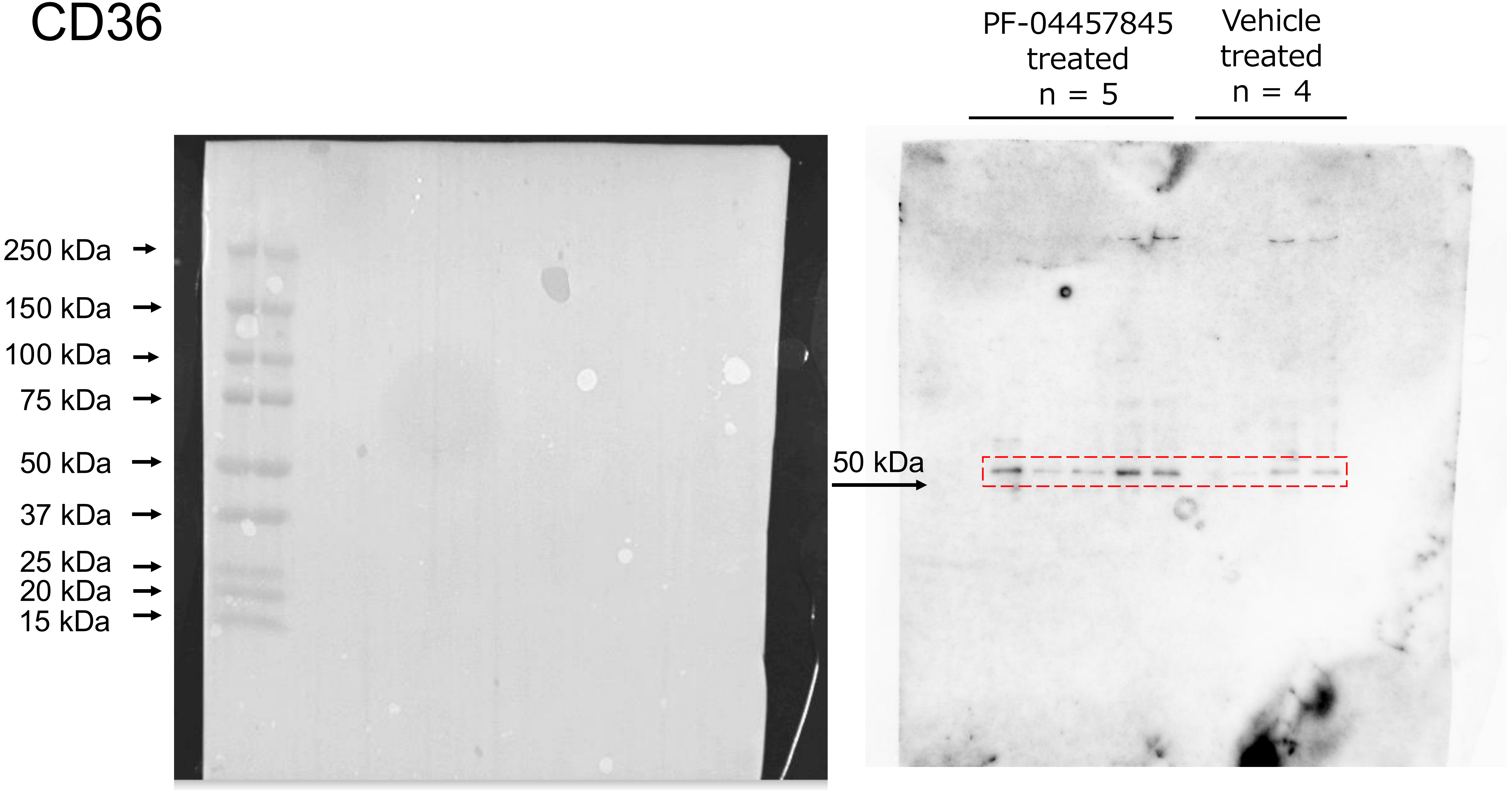

Full unedited membrane of CD36 for Figure 7D. The result is presented in reverse left to right in Figure 7D.

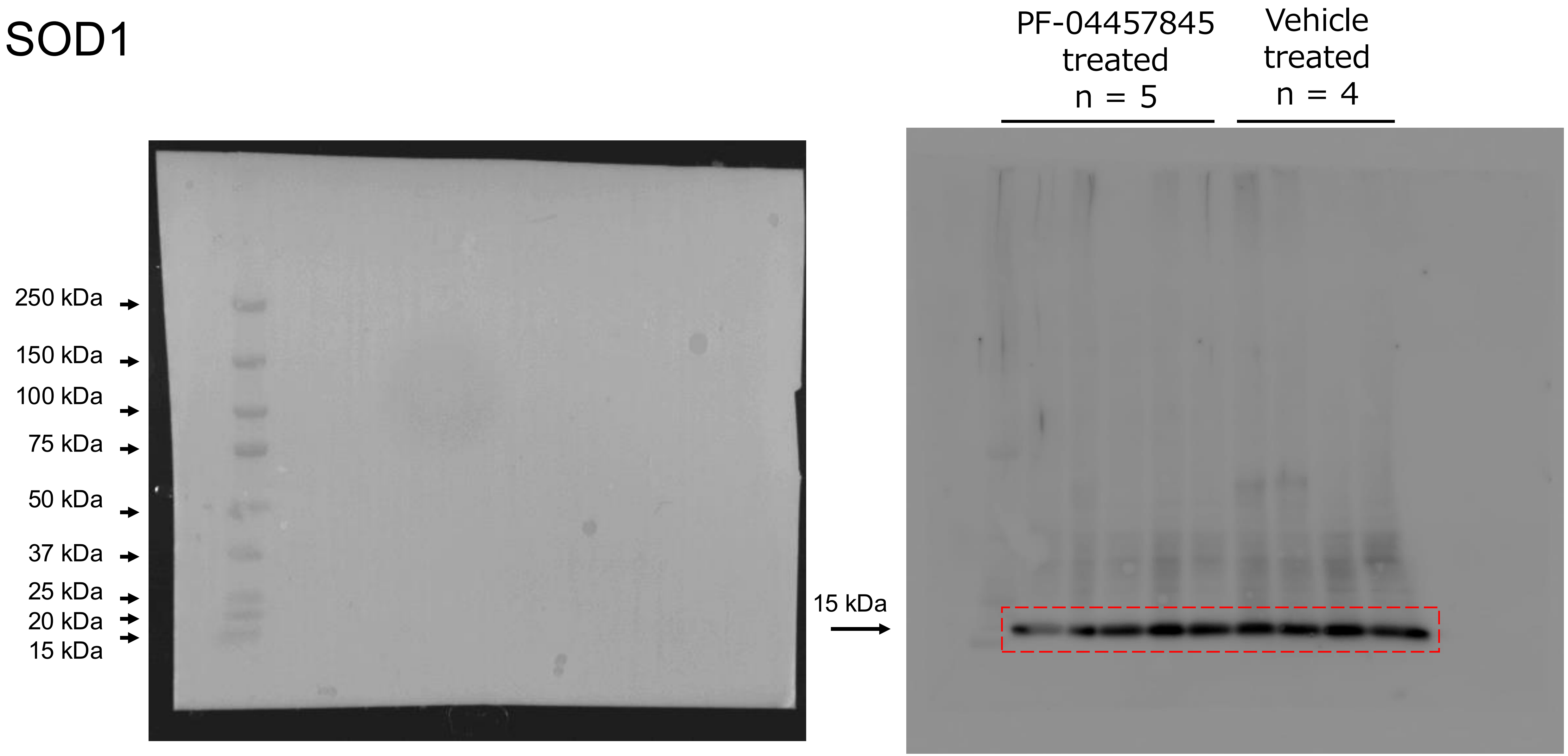

Full unedited membrane of SOD1 for Figure 7D. The result is presented in reverse left to right in Figure 7D.

CD86

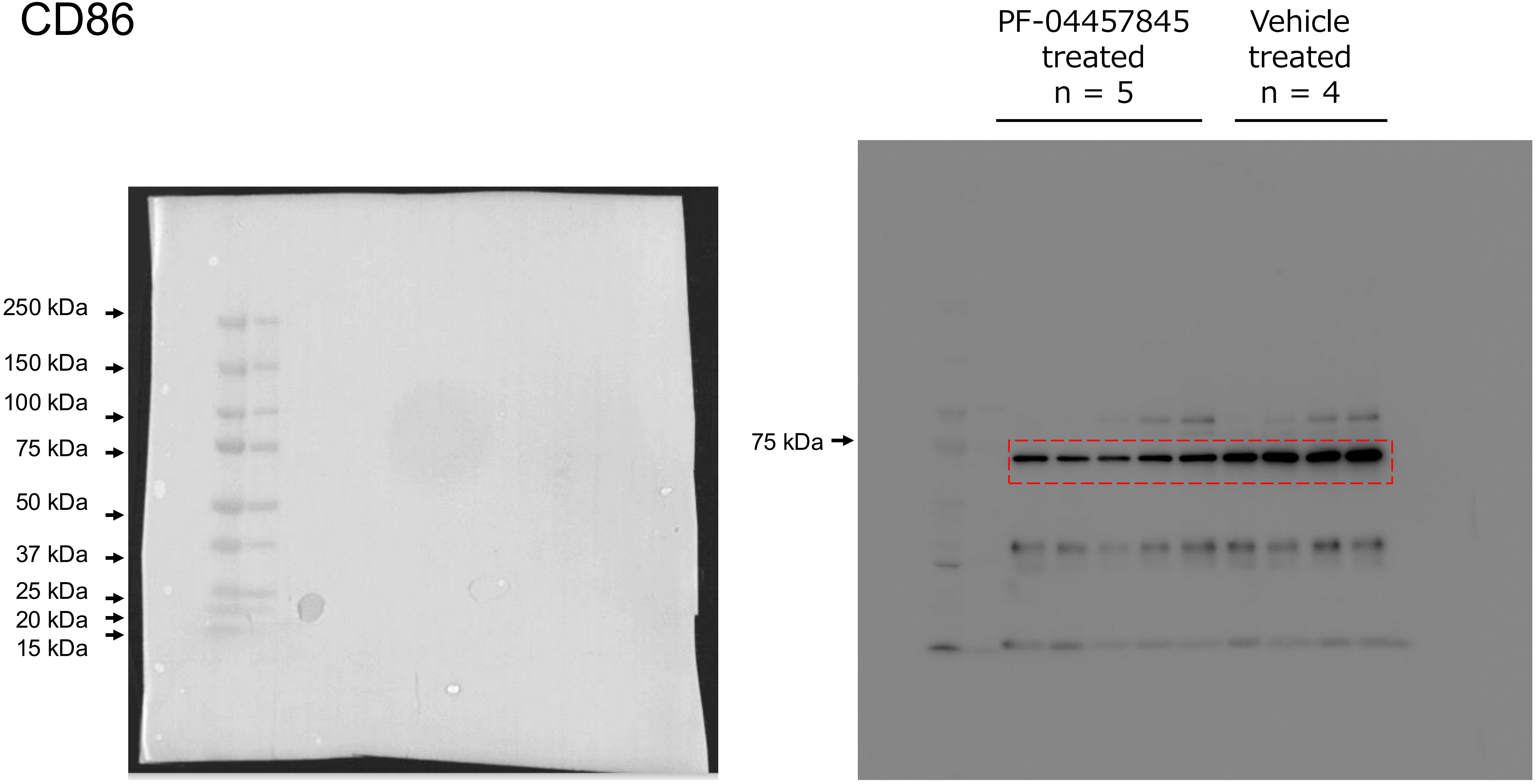

Full unedited membrane of CD86 for Figure 7D. The result is presented in reverse left to right in Figure 7D.

IBA-1

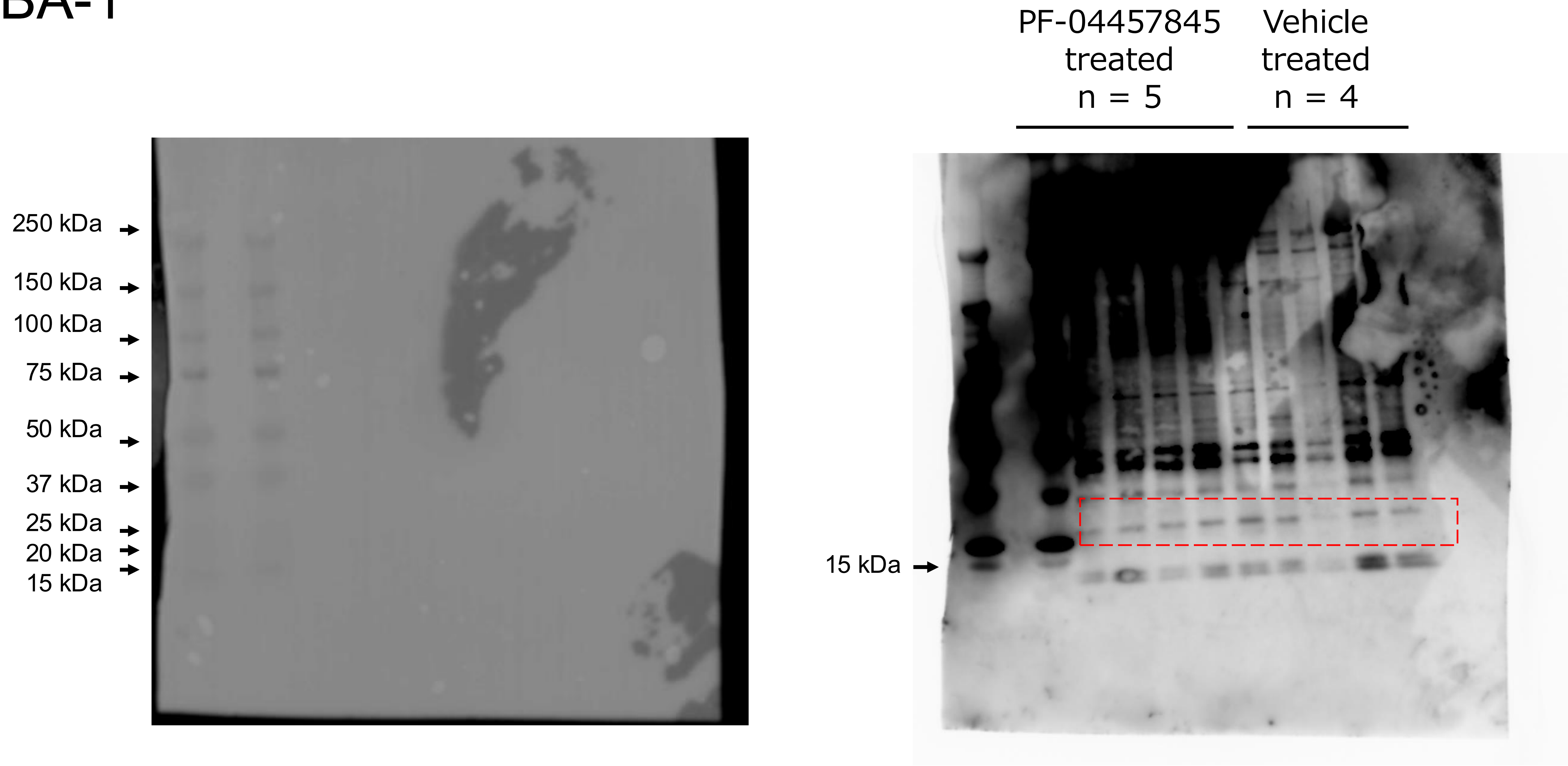

Full unedited membrane of IBA-1 for Figure 7D. The result is presented in reverse left to right in Figure 7D.

ERK1/2

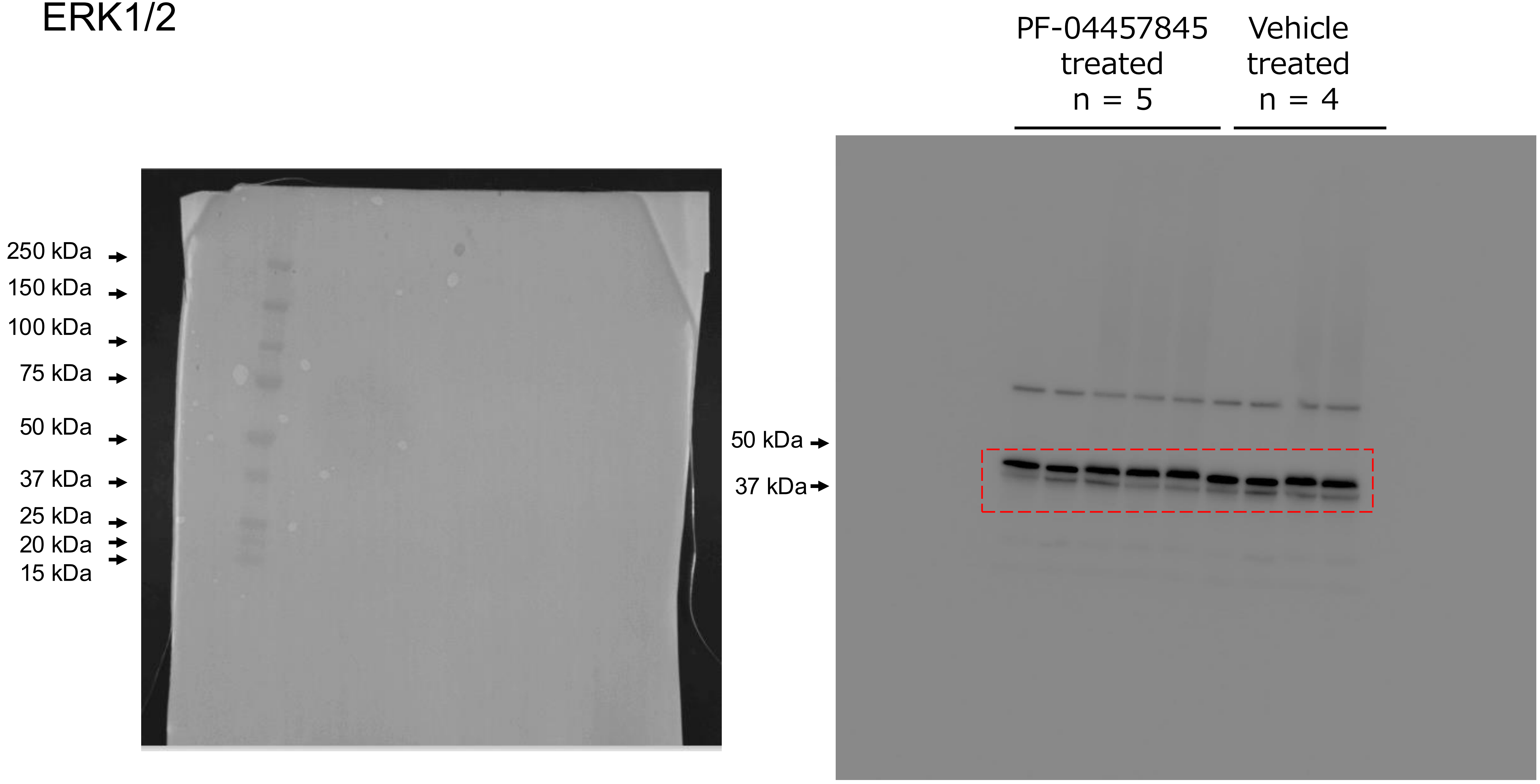

Full unedited membrane of ERK1/2 for Figure 7D. The result is presented in reverse left to right in Figure 7D.

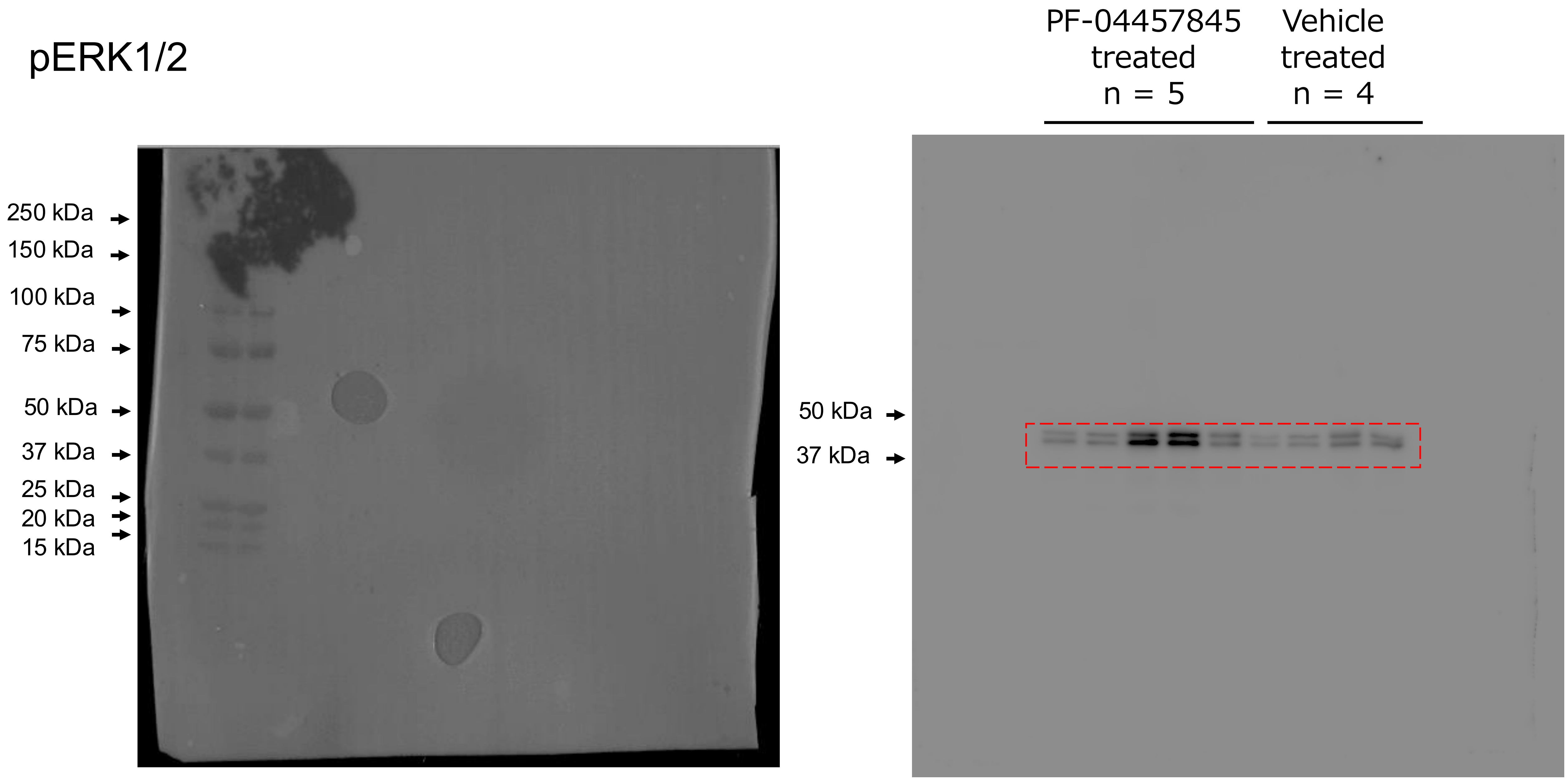

Full unedited membrane of pERK1/2 for Figure 7D. The result is presented in reverse left to right in Figure 7D.

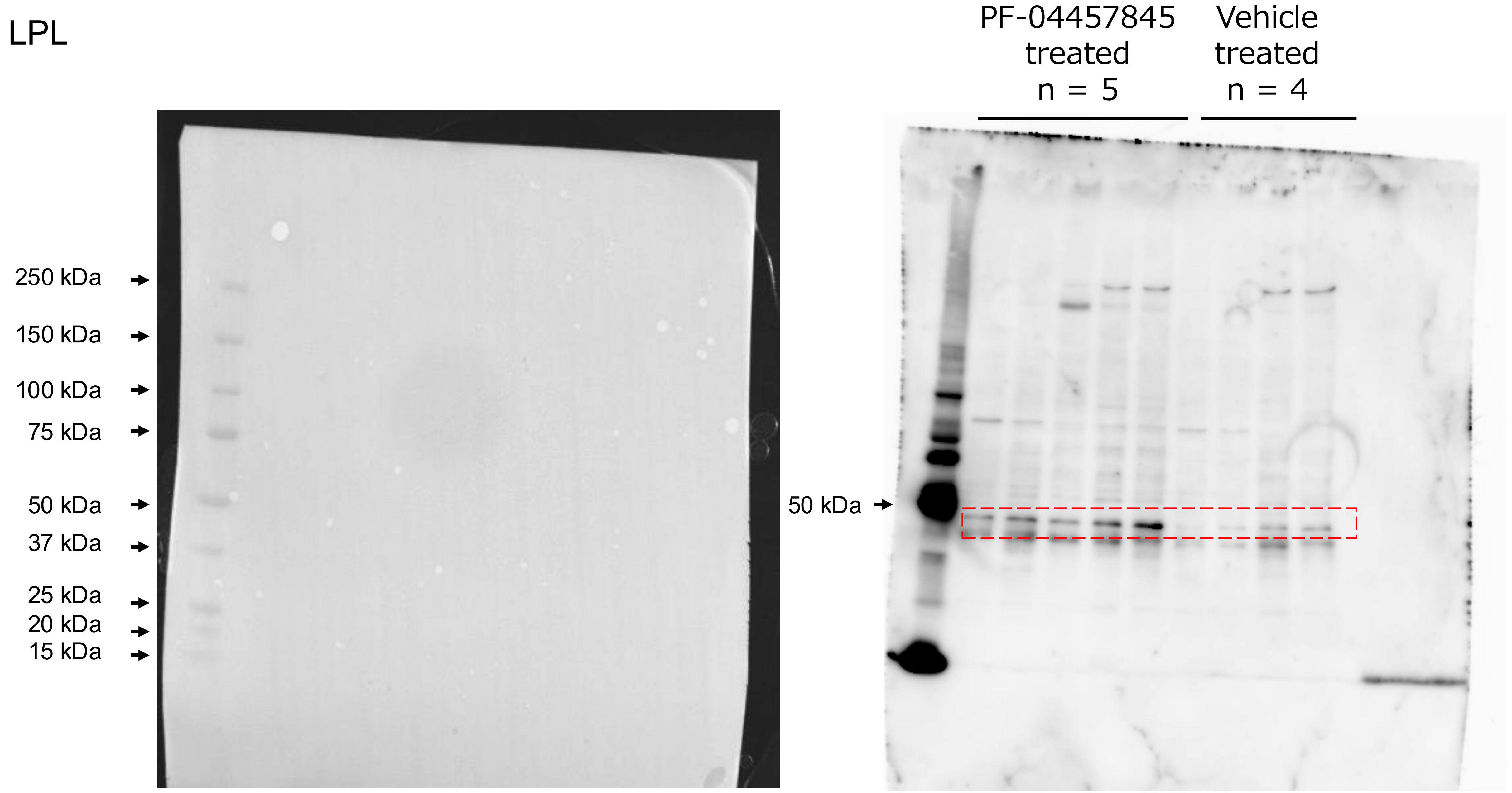

Full unedited membrane of LPL for Figure 7D. The result is presented in reverse left to right in Figure 7D.

GAPDH

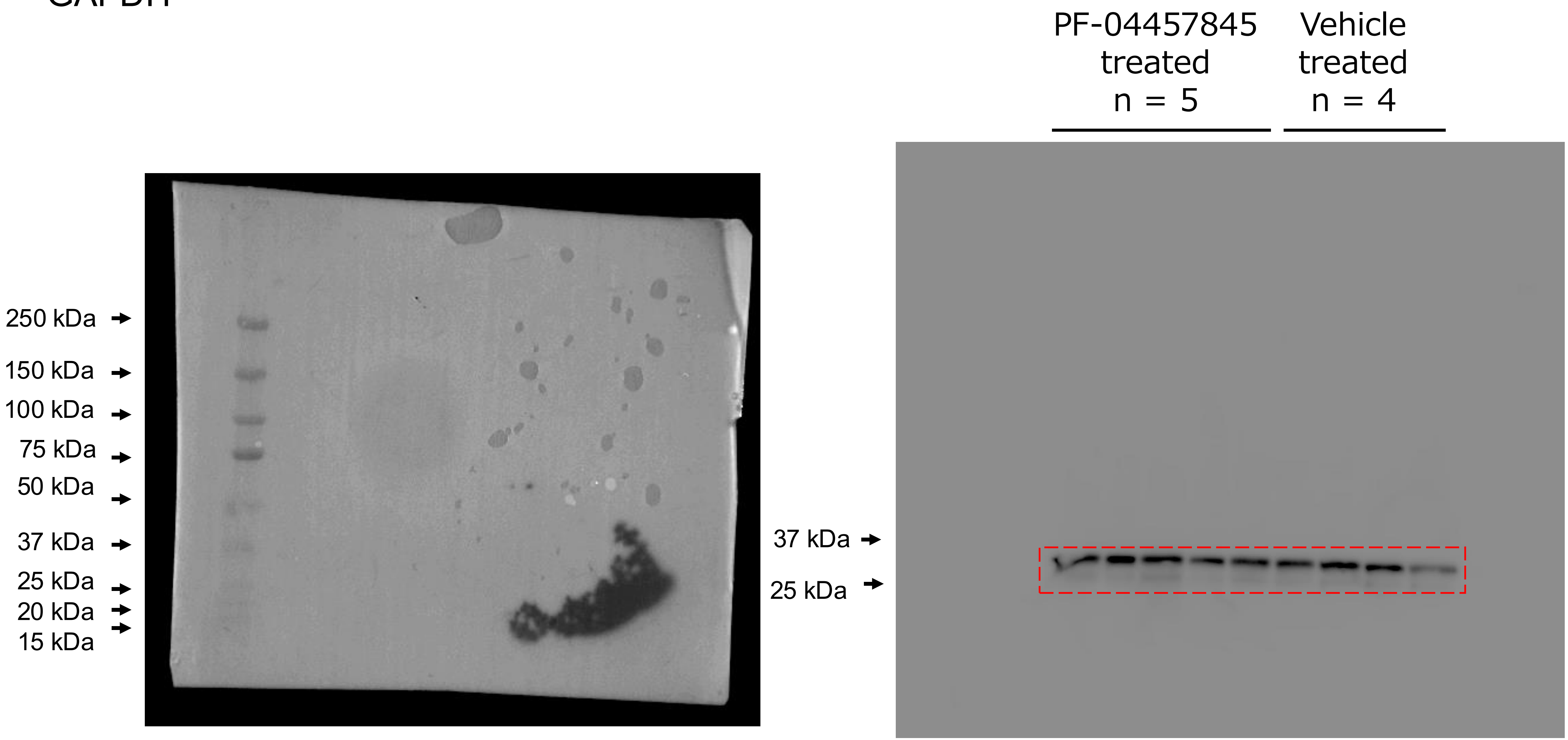

Full unedited membrane of GAPDH for Figure 7D. The result is presented in reverse left to right in Figure 7D.
